# Antimicrobial Meroterpenoids and Erythritol Derivatives Isolated from the Marine-Algal-Derived Endophytic Fungus *Penicillium chrysogenum* XNM-12

**DOI:** 10.3390/md18110578

**Published:** 2020-11-20

**Authors:** Kuo Xu, Xu-Lun Wei, Lin Xue, Zhong-Feng Zhang, Peng Zhang

**Affiliations:** 1 Tobacco Research Institute, Chinese Academy of Agricultural Sciences, Qingdao 266101, China; xukuo@caas.cn; 2 ETSONG (Qingdao) Industry Co., Ltd., Qingdao 266021, China; weixulun2020@163.com; 3 Wannan Tobacco Group Co., Ltd., Xuancheng 242000, China; xuelin-xx@163.com

**Keywords:** algal, endophytic fungus, *Penicillium*, meroterpenoids, antimicrobial activity

## Abstract

One new meroterpenoid-type alkaloid, oxalicine C (**1**), and two new erythritol derivatives, penicierythritols A (**6**) and B (**7**), together with four known meroterpenoids (**2**–**5**), were isolated from the marine algal-derived endophytic fungus *Penicillium chrysogenum* XNM-12. Their planar structures were determined by means of spectroscopic analyses, including UV, 1D and 2D NMR, and HRESIMS spectra. Their stereochemical configurations were established by comparing the experimental and calculated electronic circular dichroism (ECD) spectra for compound **1**, as well as by comparison of the optical rotations with literature data for compounds **6** and **7**. Notably, oxalicine C (**1**) represents the first example of an oxalicine alkaloid with a cleaved α-pyrone ring, whereas penicierythritols A (**6**) and B (**7**) are the first reported from the *Penicillium* species. The antimicrobial activities of compounds **1**–**7** were evaluated. Compounds **1** and **6** exhibited moderate antibacterial effects against the plant pathogen *Ralstonia solanacearum* with minimum inhibitory concentration (MIC) values of 8 and 4 μg/mL, respectively. Compound **6** also possesses moderate antifungal properties against the plant pathogen *Alternaria alternata* with a MIC value of 8 μg/mL.

## 1. Introduction

Fungal secondary metabolites of marine origin have drawn considerable attention because of their unique chemical structures and potential pharmaceutical applications [1,2,3]. Marine-derived filamentous fungi have been proven to be more prolific sources of bioactive natural products than those found in land-based systems [4,5,6,7,8,9]. Marine-derived fungi can be distributed in all marine habitats, ranging from marine plants to marine invertebrates and vertebrates and from marine sediments to deep-sea hydrothermal and cold springs [2,6,7,8]. Marine algae harbor a rich fungal endophyte community. These endophytes can harmoniously colonize the internal tissues of their hosts and produce bioactive secondary metabolites that protect their hosts in the interactional processes of symbiosis and evolution [10,11,12,13].

**Figure 1:**
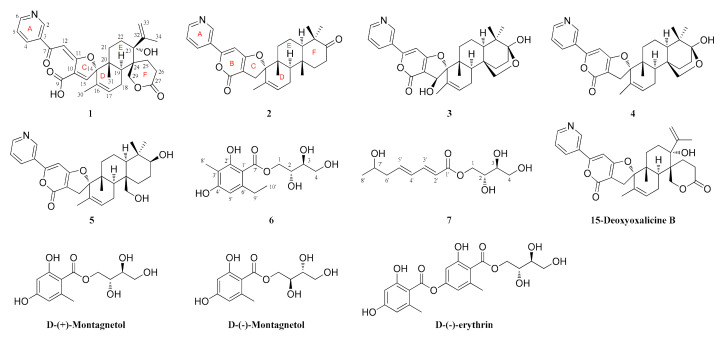
Chemical structures of the isolated compounds **1**–**7** and the previously reported analogs, 15-deoxyoxalicine B, d-(+)-montagnetol, d-(−)-montagnetol, and d-(−)-erythrin.

In our ongoing efforts to discover structurally unique and biologically active secondary metabolites from algal-derived endophytic fungi, the fungal strain *Penicillium chrysogenum* XNM-12 from the marine brown alga *Leathesia nana* (Chordariaceae) was chosen for further chemical investigation. Solid regime cultivation of *P. chrysogenum* XNM-12 led to the isolation of one new meroterpenoid-type alkaloid (**1**) and two new erythritol derivatives (**6** and **7**), as well as four known meroterpenoids (**2**–**5**) (Figure 1). The planar structures of these new compounds were determined by detailed analyses of their spectroscopic data, and their absolute configurations were assigned by comparing the experimental and calculated electronic circular dichroism (ECD) spectra and optical rotations with literature data. Compounds **1**–**5** are rare members of a group of unique natural products containing terpenoid and pyridine moieties, with approximately 20 compounds reported to date [14,15,16]. Notably, compound **1** represents the first example of an oxalicine alkaloid with a cleaved α-pyrone ring, whereas compounds **6** and **7** are reported from a *Penicillium* species for the first time. In this study, we report the isolation, structural elucidation, and biological evaluation of these compounds.

## 2. Results and Discussion

### 2.1. Structural Elucidation of New Compounds

**Figure 2:**
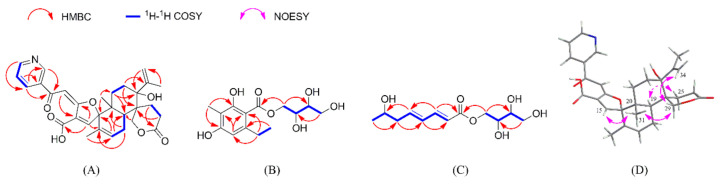
(**A**–**C**) COSY and key HMBC correlations of **1**, **6**, and **7**, respectively; (**D**) Key NOESY correlations of **1**.

Compound **1** was isolated as a white amorphous powder. Its molecular formula was determined to be C_30_H_33_NO_7_ based on its HRESIMS data (*m*/*z* 520.2330 [M + H]^+^, calcd. 520.2335), indicating 15 degrees of unsaturation. The ^1^H NMR spectrum (Table 1) revealed four signals that resonated at *δ*_H_ 8.89 (1H, s, H-2), 8.14 (1H, d, *J* = 8.0 Hz, H-4), 7.50 (1H, m, H-5), and 8.66 (1H, d, *J* = 4.5 Hz, H-6), which are typical of a two-substituted pyridine moiety [14,15,16]. In addition, its ^1^H NMR spectrum displayed signals for three olefinic protons at *δ*_H_ 7.09 (1H, s, H-12), 7.32 (1H, s, H-15), and 5.75 (1H, s, H-17), a terminal exocyclic methylene group at 5.08 (1H, s, H-33a) and 4.84 (1H, s, H-33b), one methylene group at *δ*_H_ 4.43 (2H, s, H-29), five sets of diastereopic protons (CH_2_-18, CH_2_-21, CH_2_-22, CH_2_-25, and CH_2_-26), one methine proton at *δ*_H_ 2.57 (1H, dd, *J* = 5.0, 12.0 Hz, H-19), and three methyl groups at *δ*_H_ 1.36 (3H, s, H-30), 0.94 (3H, s, H-31), and 1.75 (3H, s, H-34) (Table 1). The ^l3^C NMR data combined with the HSQC spectrum supported the above observations. The ^l3^C NMR and DEPT spectra (Table 1) revealed the presence of 30 carbon atoms, which were classified into 3 methyl, 7 methylene (including an exocyclic methylene group and an oxymethylene unit), 8 methine (including three trisubstituted olefins), and 12 quaternary (including one conjugated ketone carbonyl, two carbonyls, and five olefinic) carbons. Together with the ^1^H-^1^H COSY data (Figure 2A), the four olefinic proton resonances at *δ*_H_ 7.50 (m), 8.14 (d, 8.0), 8.66 (d, 4.5), and 8.89 (s) and the five olefinic carbon resonances at *δ*_C_ 123.5 (CH), 128.5 (C), 135.0 (CH), 148.3 (CH), and 151.4 (CH) further confirmed that a two-substituted pyridine unit was present in **1**. A pair of olefinic proton resonances at *δ*_H_ 4.84 (s) and 5.08 (s) and an aliphatic proton resonance at *δ*_H_ 1.75 (s) associated with the carbon resonances at *δ*_C_ 21.7 (CH_3_), 114.3 (CH_2_), and 150.5 (C) revealed that an isopropenyl unit also existed in the structure. These spectroscopic features indicated that **1** is closely related to the known compound 15-deoxyoxalicine B, which was previously isolated from two new species: *Penicillium decaturense* and *Penicillium thiersii* [15]. Analysis of their 1D NMR spectra suggested that the main difference between **1** and 15-deoxyoxalicine B is the central α-pyrone ring B (Figure 1). In the HMBC spectrum (Figure 2A), H-2 and H-4 showed clear correlations with the ketone carbonyl carbon (*δ*_C_ 186.4, C-7), indicating the cleavage of ring B. HMBC correlations from H-12 to C-7/C-10/C-11 and H-15 to C-9/C-11/C-16 located the remaining olefinic groups at C-12 and C-15, respectively, and established the conjugated linkage from C-7 to C-15. Therefore, **1** was identified as the rearranged analog of 15-deoxyoxalicine B. Compound **1** is considered an alkaloidal meroterpene belonging to the oxalicine family and was assigned the name oxalicine C.

**Figure 3:**
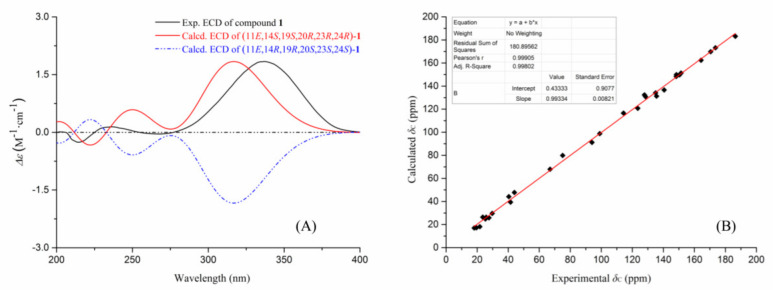
The theoretical calculations for compound **1**. (**A**) experimental and calculated ECD spectra of **1**; (**B**) correlation between the calculated and experimental ^13^C NMR data of **1**.

The stereochemistry of **1** was assigned by analyses of the NOESY data (Figure 2D) and theoretical calculations (Figure 3). In the NOESY experiment, the signal for H_3_-31 (*δ*_H_ 0.94) exhibited unambiguous NOESY correlations to H-15 (*δ*_H_ 7.32) and H_2_-29 (*δ*_H_ 4.43), whereas the signal for H-19 (*δ*_H_ 2.57) showed an obvious correlation to H-25b (*δ*_H_ 1.45). These data indicated that H_3_-31 and H-15 are located on the same face of the cyclohexene ring, and H_3_-31 has a *cis*-axial relationship with H_2_-29 in the cyclohexane ring. The absence of correlated signals for H_3_-31 with H-19 revealed that H_3_-31 and H-19 have a *trans*-diaxial relationship; thus, the cyclohexane and cyclohexene rings are *trans*-fused. An additional NOESY correlation of H_3_-34 (*δ*_H_ 1.75) with H_2_-29 requires the isopropenyl group to adopt an equatorial orientation in the E-cyclohexane ring, and it occupies the same face as H_3_-31 and H_2_-29. The configuration of the Δ^11,^^12^ olefinic bond was tentatively deduced as *E*-configuration by a biogenetic analogy to the stereochemistry of oxalicines A and B [15,16]. Based on the relative configurational assignments as described above, two optimized conformers (11*E*, 14*S*, 19*S*, 20*R*, 23*R*, and 24*R*) and (11*E*, 14*R*, 19*R*, 20*S*, 23*S*, and 24*S*) were calculated using time-dependent density functional theory (TDDFT) at the B3LYP/6-311G (d,p) and MPW1PW91/6-311+G(2d,p) levels, respectively (Supplementary Materials, Tables S1 and S2). As shown in Figure 3, the experimental ECD spectrum of **1** matched well with the calculated spectrum of the (14*S*, 19*S*, 20*R*, 23*R*, and 24*R*) isomer. The experimental ^13^C NMR data of **1** and the calculated data of this isomer also showed good linear dependence with a high *R*^2^ value and low corrected mean absolute deviation (CMAD) and corrected largest absolute deviation (CLAD) values. Thus, the absolute configuration of **1** was assigned, consistent with the stereochemistry originally assigned for oxalicines A and B [14,15,16].

In addition, another four known meroterpenoid-type alkaloids were isolated in this study. They were identified as decaturin D (**2**) [16], decaturin B (**3**) [15,16], decaturin C (**4**) [16], and decaturin F (**5**) [17] by comparison of their NMR data (Supplementary Materials, Figures S9–S20) and specific rotation values (Supplementary Materials, Table S6) with literature data. These compounds usually possess pyridinyl-α-pyrone and diterpenoid substructures, which are rare among natural products. To date, no more than 20 compounds belonging to the oxalicine and decaturin families have been reported. Suspicion might be immediately raised regarding whether oxalicine C (**1**) was a hydrolysis or dehydration artifact of 15-deoxyoxalicine (Figure 1), a known metabolite previously identified from *Penicillium* spp. [15].

Compound **6** was obtained as a white amorphous powder. It was determined to possess a molecular formula of C_14_H_20_O_7_ based on its HRESIMS data at *m*/*z* 323.1109 ([M + Na]^+^, calcd. 323.1107). Its ^1^H and ^13^C NMR data (Table 2), in accordance with the HSQC spectrum, exhibited two methyls (including an aromatic methyl at *δ*_H_ 1.93 (3H, s, H-8′) and an sp^3^ methyl at *δ*_H_ 1.10 (3H, t, *J* = 7.5 Hz, H-10′)), three sp^3^ methylenes (including two oxygenated, CH_2_-1 (*δ*_H_ 4.44, d, *J* = 11.0 Hz, H-1a; 4.26, dd, *J* = 11.0, 7.0 Hz, H-1b; *δ*_C_ 67.2) and CH_2_-4 (*δ*_H_ 3.58, m, H-4a; 3.36, m, H-4b; *δ*_C_ 63.0)), three methines (one aromatic (*δ*_H_ 6.30, s, H-5′; *δ*_C_ 109.2, C-5′) and two oxygenated (*δ*_H/C_ 3.71/69.2, H/C-2; *δ*_H/C_ 3.42/72.5, H/C-3)), five substituted aromatic carbons (*δ*_C_ 103.6, C-1′; 161.1, C-2′; 108.2, C-3′; 160.7, C-4′; 145.1, and C-6′), and one ester carboxyl carbon (*δ*_C_ 171.0, C-7′). Detailed analysis of the 1D and 2D NMR spectra indicated **6** is an erythritol derivative [18,19,20]. The butanetetraol moiety was determined by the mutual COSY and HMBC correlations from H-1 to H-4 (Figure 2B). Obvious HMBC correlations from H_2_-9′ to C-1′ and C-5′ and from H_3_-10′ to C-6′ indicated that the ethyl group is located at C-6′. Other key HMBC correlations determine the construction of the planar structure (Figure 2B). A trivial name, penicierythritol A, was assigned to this compound.

Compound **7** was also obtained as a white amorphous powder and possessed a molecular formula of C_12_H_20_O_6_ based on its HRESIMS data (*m*/*z* 283.1163 ([M + Na]^+^, calcd 283.1158). The ^13^C NMR spectrum of **7** (Table 2) showed 12 carbon resonances ascribed to one methyl, three methylenes (including two oxygenated), seven methines (including four olefinic and one oxygenated), and one ester carbonyl carbon. Its NMR spectroscopic data were very similar to **6**, especially in the upfield region, suggesting the presence of a butanetetraol moiety. However, the signals from the pentasubstituted benzene ring (from C-1′ to C-6′) in the NMR spectra of **6** were absent in **7**. Instead, four olefinic methines (*δ*_C_ 119.3, C-2′; 145.0, C-3′; 129.8, C-4′; and 142.0, C-5′) were observed in **7**. The COSY correlation from H-2′ to H_3_-8′ constructed their connection, which was also supported by their mutual HMBC correlations (Figure 2C). Compound **7** was named penicierythritol B.

Structurally, compounds **6** and **7** were characterized as two butantetraol analogs containing an acyclic vicinal diol moiety. The task of determining the configurations of such conformationally flexible systems is significantly challenging. In a previous study, *J*-based configurational analysis led to the assignment of two adjacent stereogenic carbons of an acyclic chain using a complete set of ^3^*J*_HH_ and ^2,3^*J*_HC_ values and key NOE data. Mosher′s method provides another stereochemical solution for this issue. However, the lack of a sufficient sample limited our ability to apply any of the above methods, thus compelling us to find other methods. A detailed literature survey revealed that some reported naturally occurring butantetraol analogs, such as d-(+)-montagnetol, d-(–)-montagnetol, and d-(+)-erythrin (Figure 1), were all characterized as erythritol derivatives rather than threitol derivatives [18,19,20]. In this study, compound **6** showed a relatively large ^3^*J*_HH_ value (5.5 Hz) between H-2 and H-3 in MeOH-*d*_4_ (Supplementary Materials, Figure S23), which further corresponds to a *threo* configuration [21]. Compound **6** was optically active and possessed consistently positive optical activity, ${\left[\alpha \right]}_{D}^{25}$ +19.1° (*c* 0.1, MeOH), which is similar to those of d-(+)-montagnetol, ${\left[\alpha \right]}_{D}^{25}$ +17.1° (*c* 0.38, MeOH), and d-(+)-erythrin, ${\left[\alpha \right]}_{D}^{25}$ +9.0° (*c* 0.2, MeOH), which proved that **6** adopts the (2*R*,3*S*) configuration [22,23,24]. Additionally, the butantetraol moiety of **7** was tentatively deduced to possess the same (2*R*,3*S*) configuration as that of **6** by comparison of the similar NMR data (Table 1) and positive specific rotation (${\left[\alpha \right]}_{D}^{25}$ +43.7°).

Erythritol derivatives were previously mainly reported from lichens, such as *Roccella* and *Trentepohlia* spp., which were assumed to be produced by the photobiont partner [18,19]. This is the first report of erythritol derivatives from marine-algal-associated fungi, indicating their potential chemotaxonomic significance in future studies.

### 2.2. Antimicrobial Activity of Compounds **1**–**7**

The isolated compounds **1**–**7** were evaluated for their antibacterial activity against human and plant pathogens (*Escherichia coli*, *Micrococcus luteus*, *Pseudomonas aeruginosa*, and *Ralstonia solanacearum*) and their anti-phytopathogenic activity against five pathogenic fungi (*Alternaria alternata*, *Botrytis cinerea*, *Fusarium oxysporum*, *Penicillium digitatum*, and *Valsa mali*), the results of which are listed in Table 3. The new compound **1** exhibited an obvious antibacterial effect against the human pathogens *E. coli*, *M. luteus*, and *P. aeruginosa*, as well as against the plant pathogen *R. solanacearum*, with minimum inhibitory concentration (MIC) values of 8 µg/mL. Notably, **6** demonstrated strong inhibition against *R. solanacearum* with a MIC value of 4 μg/mL, which was higher than that of the positive control chloromycetin (MIC = 8 μg/mL). In the antifungal assay, compound **6** exhibited impressive anti-phytopathogenic properties against *A. alternata* with a MIC value of 8 μg/mL.

## 3. Materials and Methods

### 3.1. General Experimental Procedures

UV spectra were obtained with a Shimadzu UV-2700 spectrophotometer (Shimadzu Corp., Kyoto, Japan). The optical rotations and ECD spectra were measured with a Jasco P-1020 digital polarimeter and a Jasco J-815-150S circular dichroism spectrometer (JASCO, Easton, MD, USA), respectively. NMR spectra were recorded on an Agilent DD2 500 MHz NMR spectrometer (Agilent Technologies, Waldbronn, Germany) using the residual solvent peak as the internal standard. HRESIMS spectra were obtained on a Waters Xevo G2-XS QTOF mass spectrometer (Waters Corp., Milford, MA, USA). Semipreparative HPLC was performed on a C18 (SunFire^®^, 10 µm, 19 × 250 mm) column using a Waters e2695 separation module equipped with a 2998 detector. Commercially available silica gel of 200–300 mesh (Qingdao Marine Chemical Co., Shandong, China) was used for open column chromatography.

### 3.2. Fungal Material

The fungal strain *P. chrysogenum* XNM-12 was isolated from the marine brown alga *Leathesia nana* (Chordariaceae), which was collected in April 2017 in Weihai, Shandong Province, China (37°31′57.58′′ N, 122°02′52.85′′ E). The strain was identified according to the ITS region of the rDNA gene sequence analysis by the Beijing Genomics Institute (Shenzhen, China). The strain was deposited in the Tobacco Research Institute of the Chinese Academy of Agricultural Sciences, Qingdao, China, with GenBank (NCBI) accession number MT075873.

### 3.3. Fermentation, Extraction, and Isolation

The fungal strain was cultivated in a malt extract medium (130.0 g/L malt extract, 0.1 g/L chloramphenicol, 15.0 g/L agar, pH 5.6 ± 0.2) under static conditions at 25 °C for 30 days. A total of 100 culture dishes (90 × 15 mm) were used in the experiment. After fermentation, the fresh mycelia with agar blocks were collected and exhaustively extracted three times with ethyl acetate (EtOAc). The filtrates were evaporated under reduced pressure to derive 8.6 g of soluble crude extract. Chromatographic separation of the extract was first performed on an open silica gel column (4 × 40 cm) eluted with petroleum ether (PE)-EtOAc. The obtained eluent was concentrated by reduced pressure at 45 °C and was separated to produce six fractions by HPLC (MeOH-H_2_O, 10−100%, 30 min, 1 mL/min), including fractions A (458.5 mg), B (213.1 mg), C (119.4 mg), D (100.6 mg), E (112.5 mg), and F (912.3 mg). These fractions were separated by reversed-phase preparative HPLC (pHPLC) using a continuous gradient of MeOH-H_2_O (60−100%, 30 min, 3 mL/min). The targeted compounds (absorption peaks) were further purified by preparative HPLC using a continuous gradient of MeCN-H_2_O (30−100%, 30 min, 3 mL/min). As a result, compound **3** (17.0 mg) was mainly isolated from fractions A and B; compound **5** (15.1 mg) was obtained from fractions B and C; compounds **2** (29.2 mg) and **4** (9.7 mg) were obtained from fraction D; compound **1** (29.2 mg) was isolated from fraction E; compounds **6** (5.2 mg) and **7** (3.9 mg) were acquired from fraction F. After freeze-drying, the above compounds were analyzed by NMR.

*Oxalicine C* (**1**): white amorphous powder; ${\left[\alpha \right]}_{D}^{25}$ +58.1° (c 0.15, MeOH); UV (MeOH) *λ*_max_ (log *ε*) 204 (4.21), 241 (4.04), 335 (3.81) nm; ^1^H and ^13^C NMR data, Table 1; HRESIMS *m*/*z* 520.2330 [M + H]^+^ (calcd. for C_30_ H_34_NO_7_, 520.2335).

*Penicierythritol A* (**6**): white amorphous powder; ${\left[\alpha \right]}_{D}^{25}$ +19.1° (c 0.10, MeOH); UV (MeOH) *λ*_max_ (log *ε*) 210 (4.15), 256 (3.94), 294 (3.71) nm; ^1^H and ^13^C NMR data, Table 2; HRESIMS *m*/*z* 323.1109 [M + Na]^+^ (calcd. for C_14_H_20_O_7_Na, 323.1107).

*Penicierythritol B* (**7**): white amorphous powder; ${\left[\alpha \right]}_{D}^{25}$ +43.7° (c 0.08, MeOH); UV (MeOH) *λ*_max_ (log *ε*) 223 (3.87) nm; ^1^H and ^13^C NMR data, Table 2; HRESIMS *m*/*z* 283.1163 [M + Na]^+^ (calcd. for C_12_H_20_O_6_Na, 283.1158).

### 3.4. Antimicrobial Assay

The antimicrobial activity of the isolated compounds was determined in 96-well microtitration plates using a modified method previously described in the literature [25]. The procedure is detailed in the Supplementary Materials (Page S24). Three human pathogens, *E. coli*, *M. luteus*, and *P. aeruginosa*, one bacterial plant pathogen *R. solanacearum*, and five fungal plant pathogens *A. alternata*, *B. cinerea*, *F. oxysporum*, *P. digitatum*, and *V. mali* were chosen as the test strains. All of the pathogens were purchased from Qingdao Agricultural University (Qingdao, China). The minimum inhibitory concentration (MIC) values were used to indicate the lowest concentration of the tested compound that limited visible microbial growth in the microtitration plates. Chloromycetin and prochloraz were used as positive controls for the antibacterial and antifungal assays, respectively.

## 4. Conclusions

*Penicillium* species have played an important role in drug development throughout human history. No example is more classical than the discovery of the penicillin molecule. Secondary metabolites produced by *Penicillium* species have received a remarkable boost given their intriguing structures and potential pharmaceutical exploitation. Marine-derived endophytic fungi are considered to be a prolific source of bioactive secondary metabolites with prominent agricultural applications. In this study, one new meroterpenoid-type alkaloid, oxalicine C (**1**), and two new erythritol derivatives, penicierythritols A (**6**) and B (**7**), were isolated from the marine-algal-derived endophytic fungus *Penicillium chrysogenum* XNM-12. The newly discovered oxalicine C (**1**) represents the first example of an oxalicine alkaloid with a cleaved α-pyrone ring, increasing the structural diversity of oxalicine analogs. Additionally, penicierythritols A (**6**) and B (**7**) are reported from a *Penicillium* species for the first time. The antimicrobial activities of the isolated compounds were evaluated. Compounds **1** and **6** exhibited moderate antibacterial effects against the plant pathogen *Ralstonia solanacearum* with MIC values of 8 and 4 μg/mL, respectively, whereas the positive control chloramphenicol had an MIC value of 8 μg/mL. Compound **6** also exhibited moderate antifungal property against the plant pathogen *A. alternata* with an MIC value of 8 μg/mL. The findings may provide further proof that marine natural products are promising candidates for agrochemical discovery.

## Figures and Tables

**Table 1:** ^1^H (500 MHz, *δ* in ppm, *J* in Hz) and ^13^C NMR (125 MHz) data of compound **1** in DMSO-*d*_6_.

Position	*δ*_H_ (Mult, *J* in Hz)	*δ*_C_, Type	Position	*δ*_H_ (Mult, *J* in Hz)	*δ*_C_, Type
2	8.89 (s)	148.3, CH	21a	1.89 (t, 12.5)	25.3, CH_2_
3		128.5, C	21b	0.77 (d, 12.5)	
4	8.14 (d, 8.0)	135.0, CH	22a	2.18 (t, 14.0)	27.6, CH_2_
5	7.50 (m)	123.5, CH	22b	1.31 (d, 14.0)	
6	8.66 (d, 4.5)	151.4, CH	23		75.1, C
7		186.4, C	24		44.0, C
9		164.3, C	25a	2.27 (overlap)	25.8, CH_2_
10		140.5, C	25b	1.45 (d, 15.5)	
11		170.5, C	26a	2.42 (dt, 14.0, 5.0)	29.7, CH_2_
12	7.09 (s)	94.0, CH	26b	2.20 (overlap)	
14		99.0, C	27		173.6, C
15	7.32 (s)	148.4, CH	29	4.43 (s)	67.0, CH_2_
16		135.6, C	30	1.36 (s)	19.5, CH_3_
17	5.75 (s)	127.8, CH	31	0.94 (s)	18.1, CH_3_
18a	2.29 (overlap)	23.6, CH_2_	32		150.5, C
18b	2.06 (d, 18.0)		33a	5.08 (s)	114.3, CH_2_
19	2.57 (dd, 5.0, 12.0)	41.5, CH	33b	4.84 (s)	
20		40.4, C	34	1.75 (s)	21.7, CH_3_

**Table 2:** ^1^H (500 MHz) and ^13^C NMR (125 MHz) data of compounds **6** and **7** in DMSO-*d*_6_.

No.	Compound 6		Compound 7	
	*δ*_H_ (mult, *J* in Hz)	*δ*_C_, type	*δ*_H_ (mult, *J* in Hz)	*δ*_C_, type
1a	4.44 (d, 11.0)	67.2, CH_2_	4.24 (d, 11.0)	66.2, CH_2_
1b	4.26 (dd, 11.0, 7.0)		4.01 (dd, 11.0, 7.5)	
2	3.71 (m)	69.2, CH	3.59 (m)	69.4, CH
3	3.42 (overlap)	72.5, CH	3.38 (overlap)	72.3, CH
4a	3.58 (m)	63.0, CH_2_	3.55 (m)	63.1, CH_2_
4b	3.36 (overlap)		3.39 (overlap)	
1′		103.6, C		166.5, C
2′		161.1, C	5.88 (d, 15.5)	119.3, CH
3′		108.2, C	7.24 (dd, 15.5, 10.0)	145.0, CH
4′		160.7, C	6.27 (overlap)	129.8, CH
5′	6.30 (s)	109.2, CH	6.25 (overlap)	142.0, CH
6′		145.1, C	2.22 (m)	42.6, CH_2_
7′		171.0, C	3.70 (m)	65.6, CH
8′	1.93 (s)	8.2, CH_3_	1.05 (d, 6.0)	23.3, CH_3_
9′	2.78 (m)	28.6, CH_2_		
10′	1.10 (t, 7.5)	16.2, CH_3_		

**Table 3:** Minimum inhibitory concentration (MIC) values of compounds **1**–**7** against pathogenic microorganisms (µg/mL).

Strains		MIC (µg/mL)								
		1	2	3	4	5	6	7	Ch ^a^	Pr ^b^
Bacteria	*E. coli*	8	>64	32	>64	>64	16	32	1	–
	*M. luteus*	8	>64	16	>64	32	8	16	1	
	*P. aeruginosa*	16	32	>64	>64	16	8	>64	2	
	*R. solanacearum*	8	>64	32	32	16	4	>64	8	
Fungi	*A. alternata*	>64	>64	32	32	>64	8	>64	–	16
	*B. cinerea*	32	>64	16	32	32	16	>64		8
	*F. oxysporum*	>64	>64	16	>64	32	32	32		8
	*P. digitatum*	32	>64	>64	16	>64	32	16		16
	*V. mali*	16	>64	>64	16	32	16	16		4

^a^ chloramphenicol; ^b^ prochloraz.

## Supplementary Materials

The following are available online at https://www.mdpi.com/1660-3397/18/11/578/s1. Figures S1–S35 (Pages S3–S20): HRESIMS and NMR spectra of compounds **1**–**7**; Tables S1 and S2 (Page S22): the theoretical ^13^C NMR data of compound **1**; Tables S3–S5 (Pages S25 and S26): the experimental ^1^H, ^13^C NMR, COSY, and HMBC data of compounds **1**, **6**, and **7**; Table S6 (Page S27): the specific rotations of compounds 2−5.
