# Supplementary material for: Antimicrobial Meroterpenoids and Erythritol Derivatives Isolated from the Marine-Algal-Derived Endophytic Fungus Penicillium chrysogenum XNM-12
Source: Mar Drugs. 2020 Nov 20;18(11):578. doi: 10.3390/md18110578 (PMC7699931; doi:10.3390/md18110578)
Supplement: Supplementary file 1 [file marinedrugs-18-00578-s001.pdf]

Supplementary Materials

Antimicrobial Meroterpenoids and Erythritol Derivatives Isolated from the Marine Algal-Derived Endophytic Fungus *Penicillium chrysogenum* XNM-12

Kuo Xu [1], Xu-Lun Wei [2], Lin Xue [3], Zhong-Feng Zhang [1,*] and Peng Zhang [1,*]

[1] Tobacco Research Institute of Chinese Academy of Agricultural Sciences, Qingdao 266101, China; xukuo@caas.cn (K.X.)

[2] ETSONG (Qingdao) Industry Co., Ltd., Qingdao 266021, China; weixulun2020@163.com (X.-L.W.)

[3] Wannan Tobacco Group Co., Ltd., Xuancheng 242000, China; xuelin-xx@163.com (L.X.)

* Correspondence: zhangpeng@caas.cn (P.Z.), zhangzhongfeng@caas.cn (Z.-F.Z.); Tel.: +86-532-66715079 (P.Z.), +86-532-88702239 (Z.-F.Z.)

Table of Contents

NO.	Contents	Page
**Figure S1**	The ECD spectrum of **1** in MeOH	S3
**Figure S2**	The HR-ESI-MS data of **1**	S3
**Figure S3**	The ^{1}H NMR spectrum (500 MHz) of **1** in DMSO-d_6	S4
**Figure S4**	The ^{13}C NMR and DEPT spectrum (125 MHz) of **1** in DMSO-d_6	S4
**Figure S5**	The ^{1}H-^{1}HCOSY spectrum (500 MHz) of **1** in DMSO-d_6	S5
**Figure S6**	The HSQC spectrum (500 MHz) of **1** in DMSO-d_6	S5
**Figure S7**	The HMBC spectrum (500 MHz) of **1** in DMSO-d_6	S6
**Figure S8**	The NOESY spectrum (500 MHz) of **1** in DMSO-d_6	S6
**Figure S9**	The HR-ESI-MS data of **2**	S7
**Figure S10**	The ^{1}H NMR spectrum (500 MHz) of **2** in DMSO-d_6	S7
**Figure S11**	The ^{13}C NMR and DEPT spectrum (125 MHz) of **2** in DMSO-d_6	S8
**Figure S12**	The HR-ESI-MS data of **3**	S8
**Figure S13**	The ^{1}H NMR spectrum (500 MHz) of **3** in DMSO-d_6	S9
**Figure S14**	The ^{13}C NMR and DEPT spectrum (125 MHz) of **3** in DMSO-d_6	S9
**Figure S15**	The HR-ESI-MS data of **4**	S10
**Figure S16**	The ^{1}H NMR spectrum (500 MHz) of **4** in DMSO-d_6	S10
**Figure S17**	The ^{13}C NMR and DEPT spectrum (125 MHz) of **4** in DMSO-d_6	S11
**Figure S18**	The HR-ESI-MS data of **5**	S11
**Figure S19**	The ^{1}H NMR spectrum (500 MHz) of **5** in DMSO-d_6	S12
**Figure S20**	The ^{13}C NMR and DEPT spectrum (125 MHz) of **5** in DMSO-d_6	S12
**Figure S21**	The HR-ESI-MS data of **6**	S13

**Figure S22**	The ^{1}H NMR spectrum (500 MHz) of **6** in DMSO-d_6	S13
**Figure S23**	The ^{1}H NMR spectrum (500 MHz) of **6** in MeOH-d_4	S14
**Figure S24**	The ^{13}C NMR and DEPT spectrum (125 MHz) of **6** in DMSO-d_6	S14
**Figure S25**	The ^{1}H-^{1}H COSY spectrum (500 MHz) of **6** in DMSO-d_6	S15
**Figure S26**	The HSQC spectrum (500 MHz) of **6** in DMSO-d_6	S15
**Figure S27**	The HMBC spectrum (500 MHz) of **6** in DMSO-d_6	S16
**Figure S28**	The HPLC spectrum of **6**	S16
**Figure S29**	The HR-ESI-MS data of **7**	S17
**Figure S30**	The ^{1}H NMR spectrum (500 MHz) of **7** in DMSO-d_6	S17
**Figure S31**	The ^{13}C NMR and DEPT spectrum (125 MHz) of **7** in DMSO-d_6	S18
**Figure S32**	The ^{1}H-^{1}H COSY spectrum (500 MHz) of **7** in DMSO-d_6	S18
**Figure S33**	The HSQC spectrum (500 MHz) of **7** in DMSO-d_6	S19
**Figure S34**	The HMBC spectrum (500 MHz) of **7** in DMSO-d_6	S19
**Figure S35**	The HPLC spectrum of **7**	S20
−	Theoretical calculations of compound **1**	S21
**Table S1**	The selected conformers of **1** with Boltzmann distribution over 1%	S22
**Table S2**	Experimental and calculated ^{13}C-NMR chemical shifts of **1**	S22
−	Antimicrobial assay	S24
**Table S3**	^{1}H, ^{13}C NMR, COSY and HMBC data of compound **1** in DMSO-d_6	S25
**Table S4**	^{1}H, ^{13}C NMR, COSY and HMBC data of compound **6** in DMSO-d_6	S26
**Table S5**	^{1}H, ^{13}C NMR, COSY and HMBC data of compound **7** in DMSO-d_6.	S26
**Table S6**	The experimental and reported specific rotations of compounds **2**−**5**.	S27

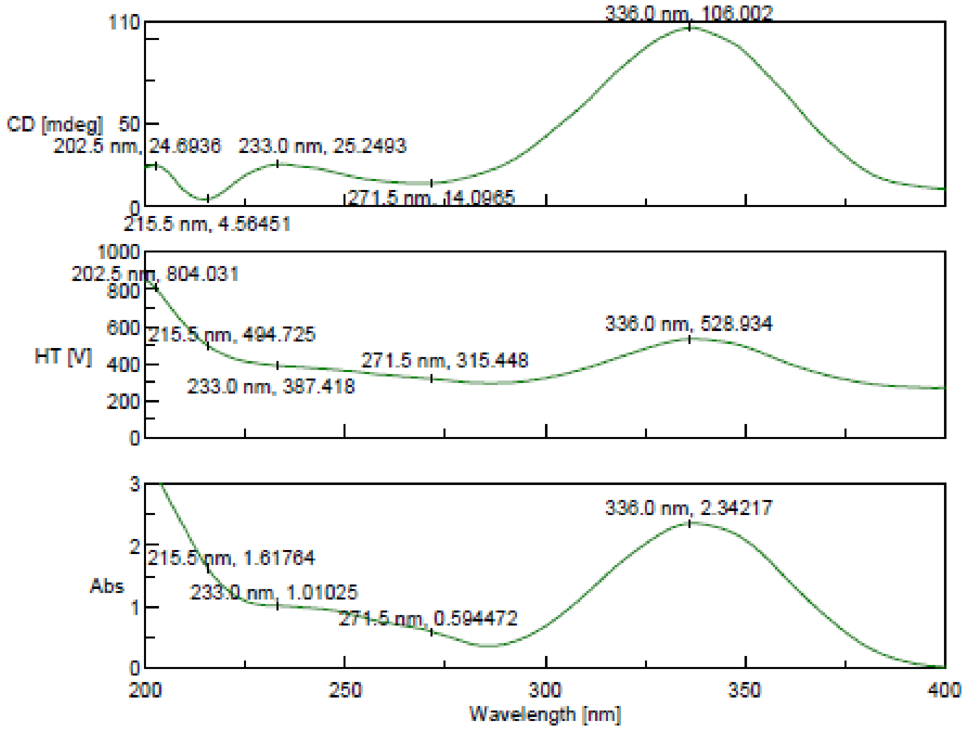

Figure S1. The ECD spectrum of **1** in MeOH

Elemental Composition Report **Page 1**

Single Mass Analysis
Tolerance = 1.0 mDa / DBE: min = -1.5, max = 50.0
Element prediction: Off
Number of isotope peaks used for i-FIT = 3

Monoisotopic Mass, Even Electron Ions
697 formula(e) evaluated with 1 results within limits (all results (up to 1000) for each mass)
Elements Used:
C: 0-50 H: 0-100 N: 0-10 O: 0-10
XK
JNP-12 180 (3.016)

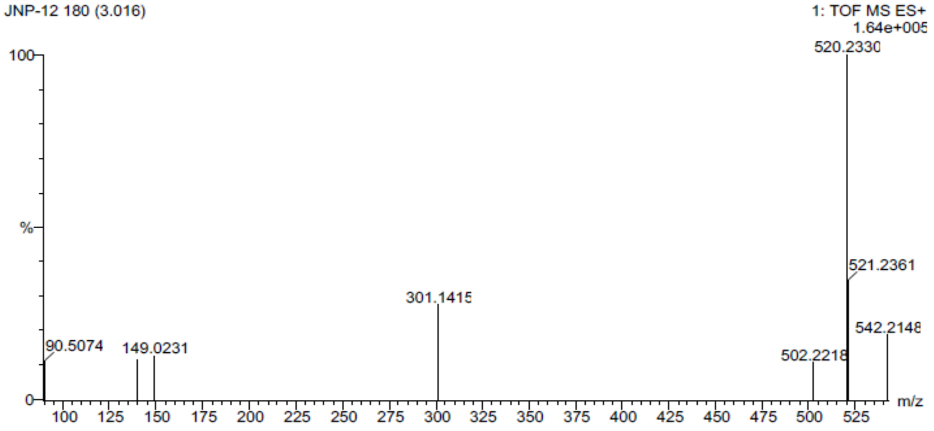

Minimum: -1.5
Maximum: 1.0 1.0 50.0

Mass	Calc. Mass	mDa	PPM	DBE	i-FIT	Norm	Conf(%)	Formula
520.2330	520.2335	-0.5	-1.0	14.5	36.3	n/a	n/a	C30 H34 N O7

Figure S2. The HR-ESI-MS data of **1**

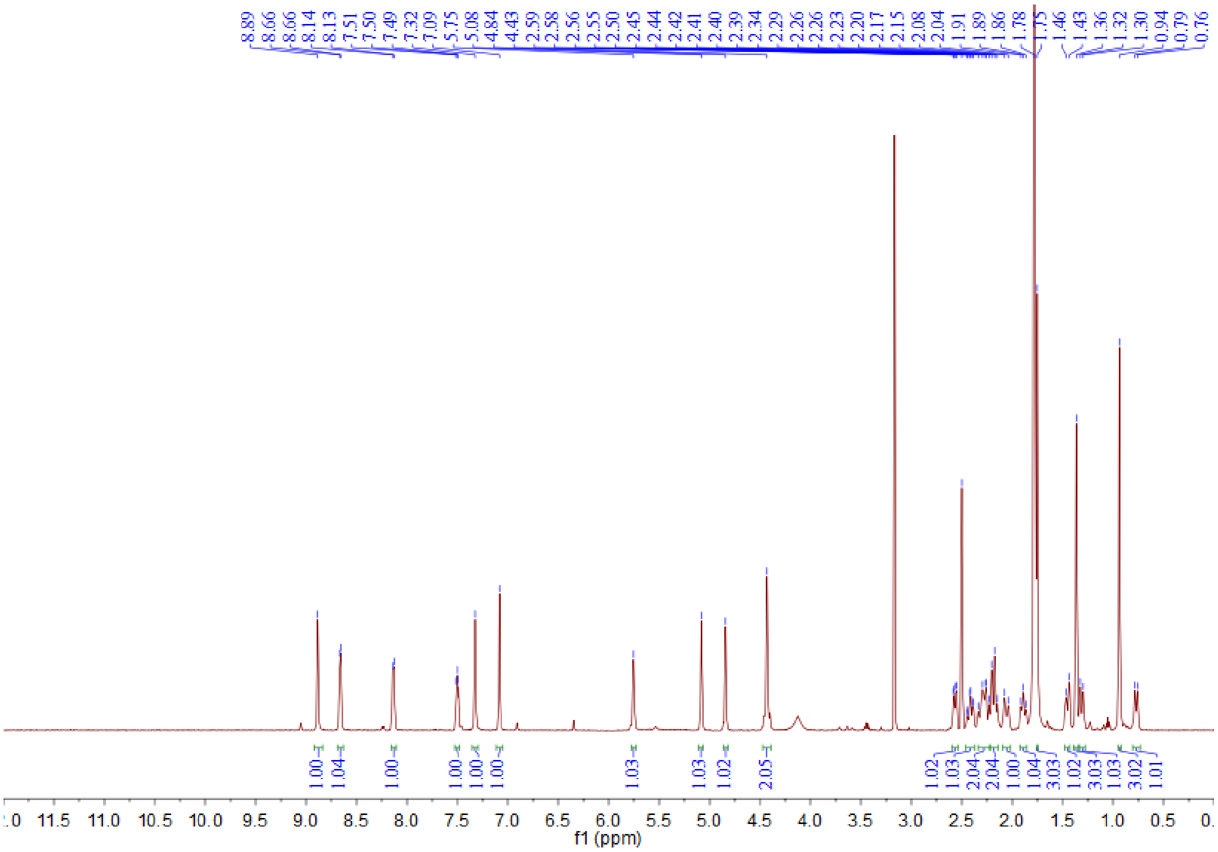

Figure S3. The ^{1}H NMR spectrum (500 MHz) of **1** in DMSO-d_6

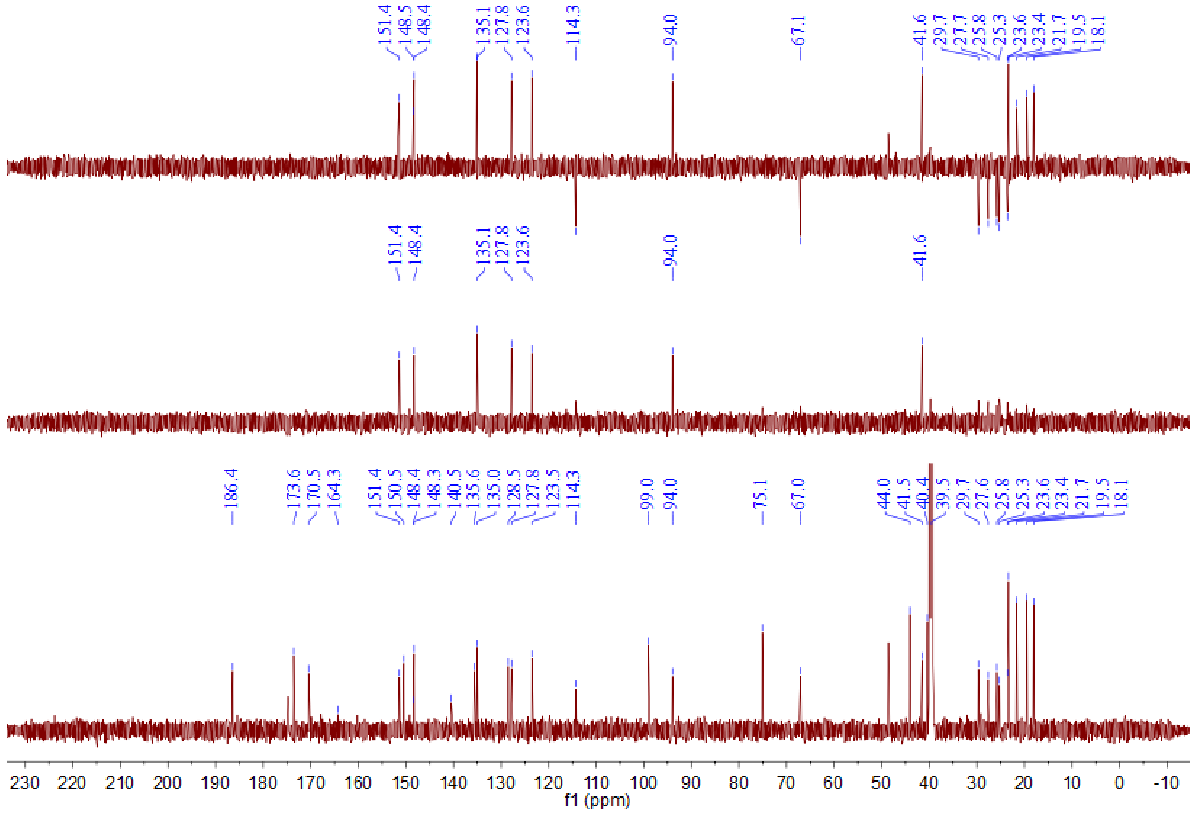

Figure S4. The ^{13}C NMR and DEPT spectrum (125 MHz) of **1** in DMSO-d_6

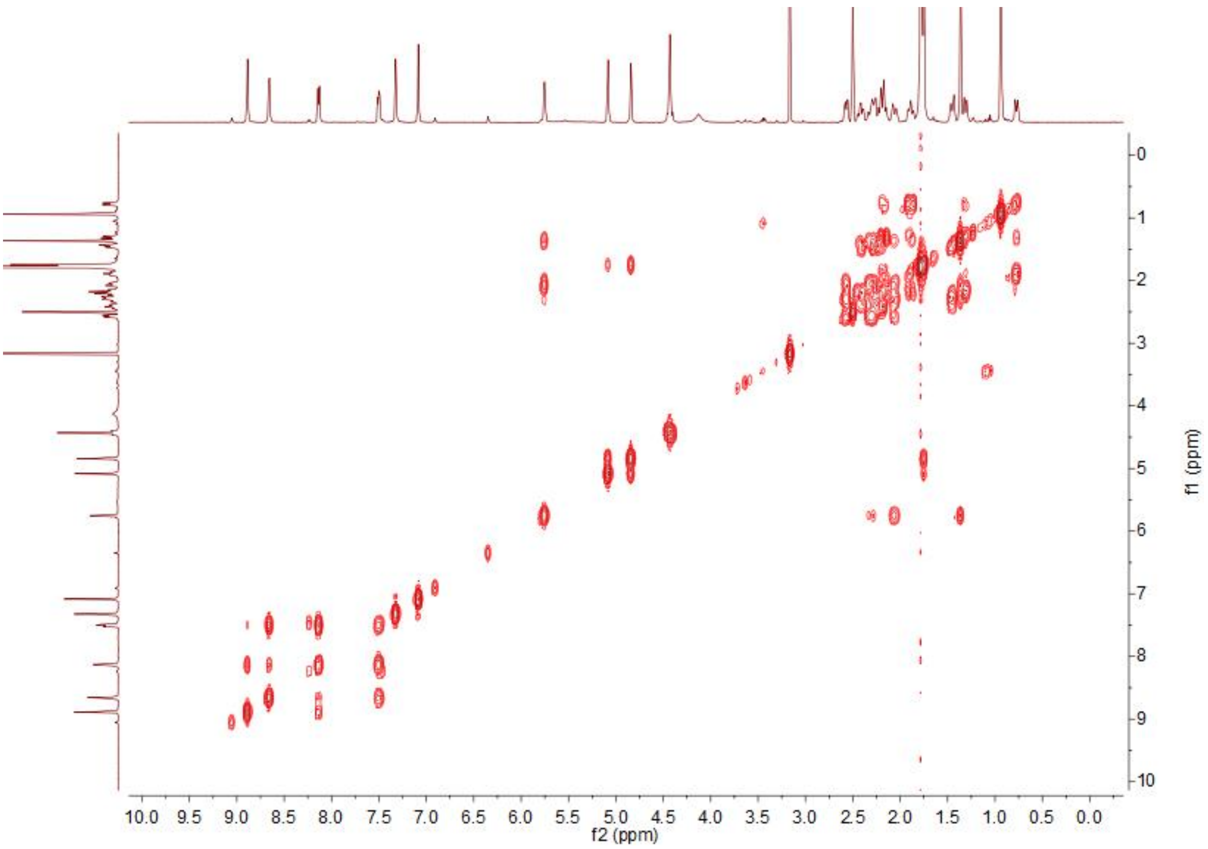

Figure S5. The ^{1}H-^{1}HCOSY spectrum (500 MHz) of **1** in DMSO-d_6

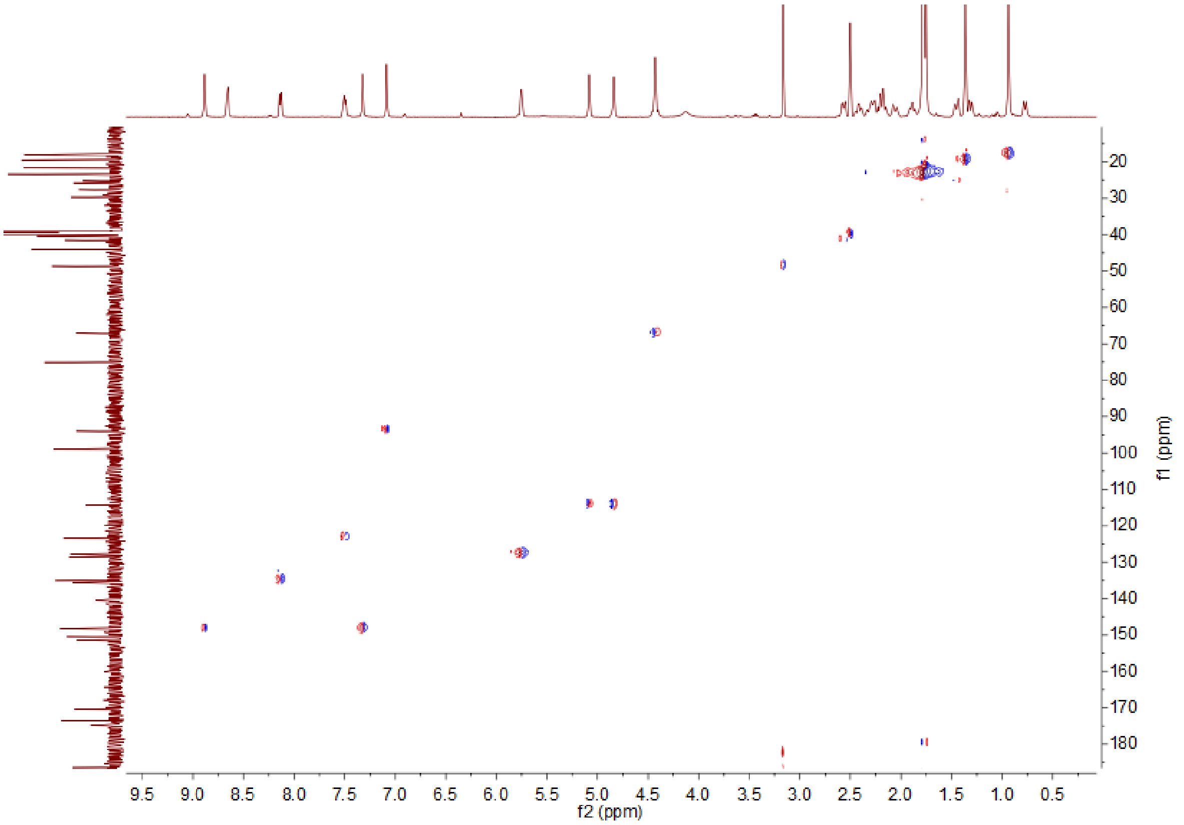

Figure S6. The HSQC spectrum (500 MHz) of **1** in DMSO-d_6

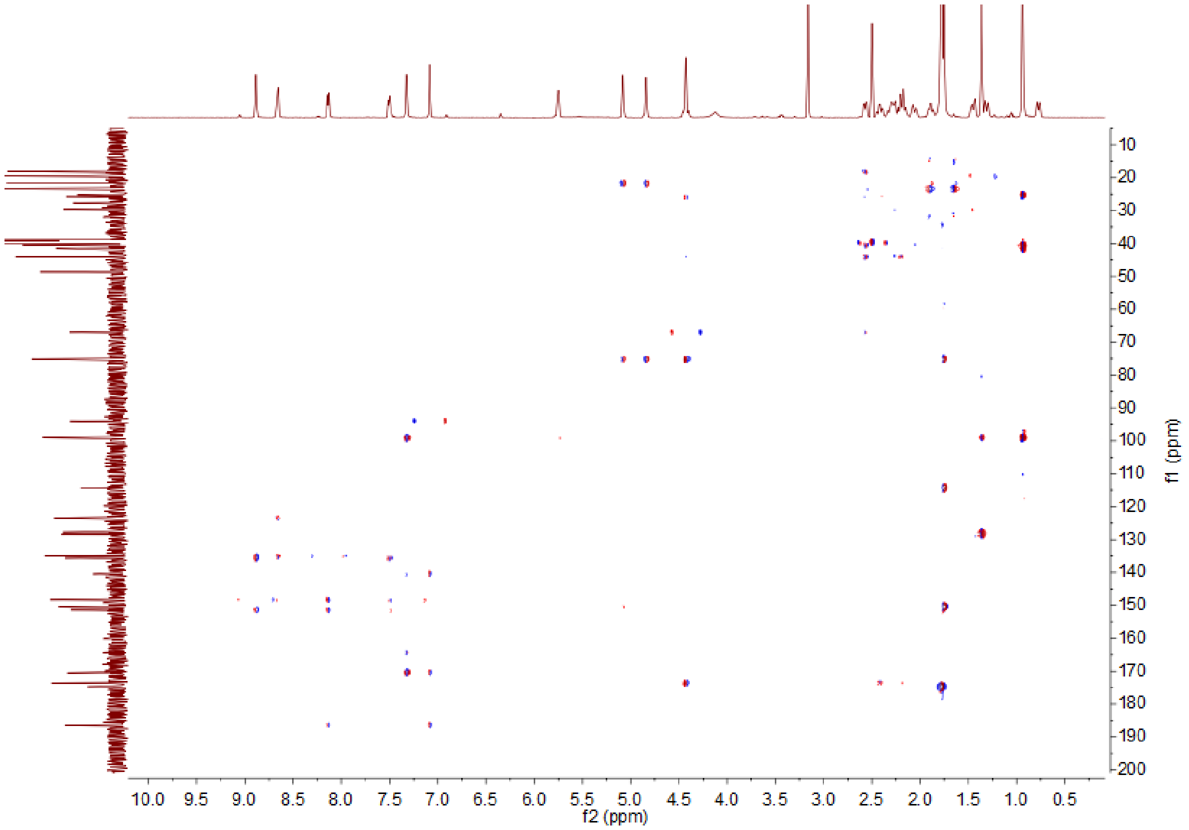

Figure S7. The HMBC spectrum (500 MHz) of **1** in DMSO-d_6

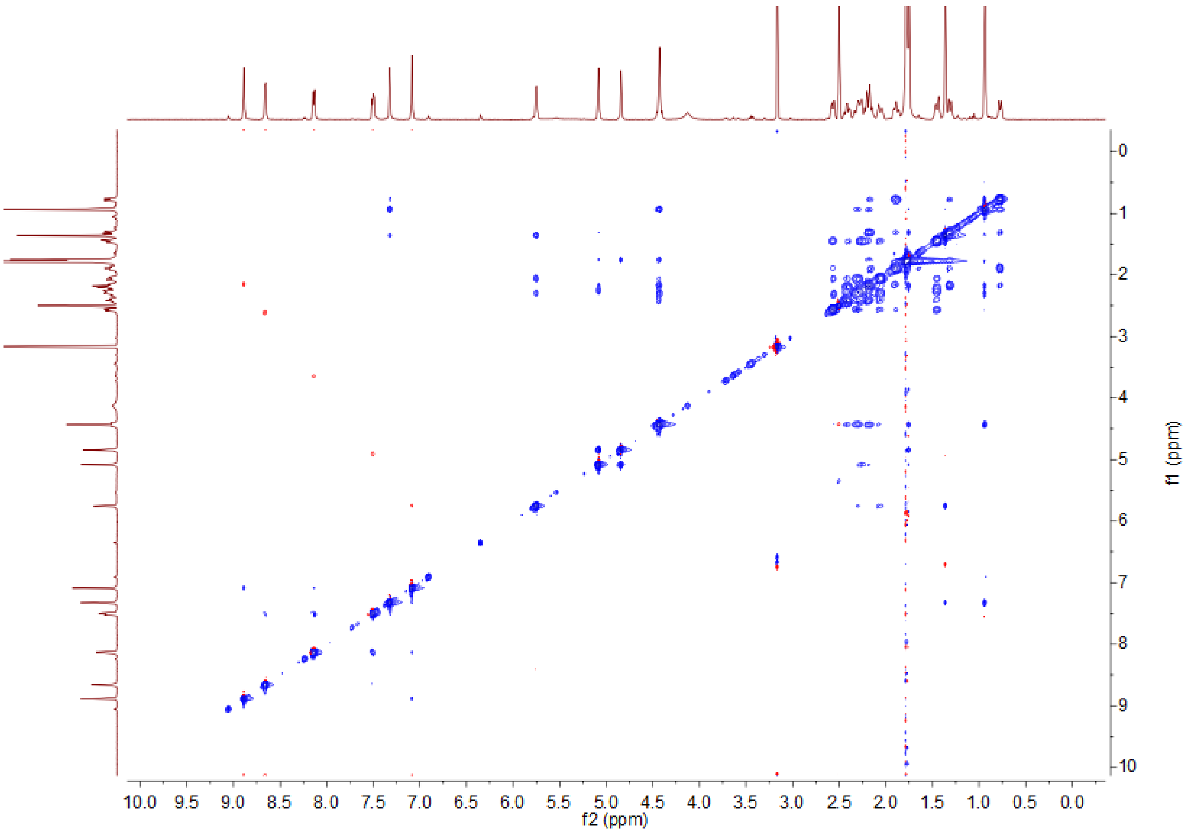

Figure S8. The NOESY spectrum (500 MHz) of **1** in DMSO-d_6

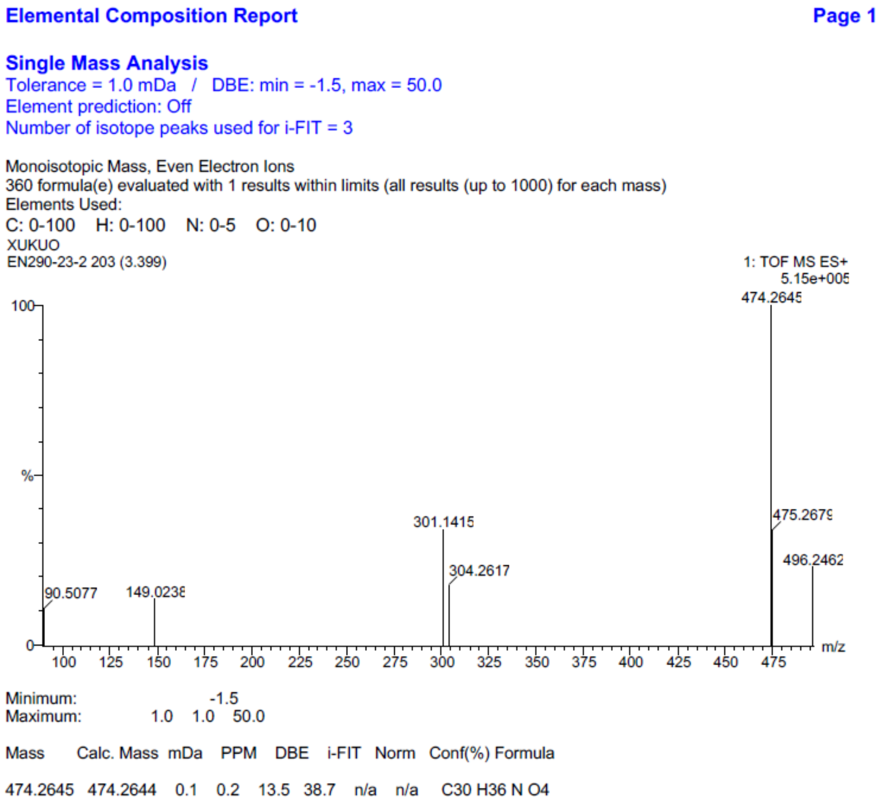

Figure S9. The HR-ESI-MS data of **2**

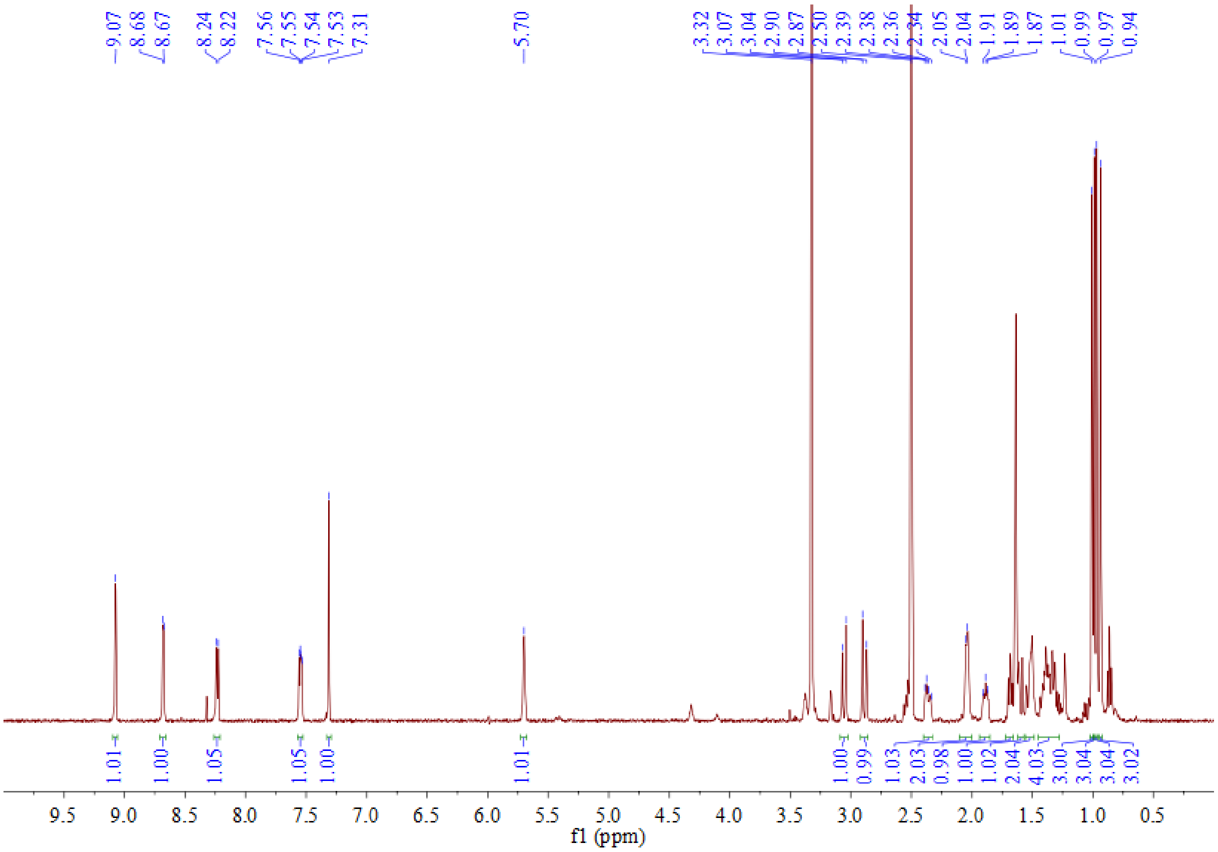

Figure S10. The ^{1}H NMR spectrum (500 MHz) of **2** in DMSO-d_6

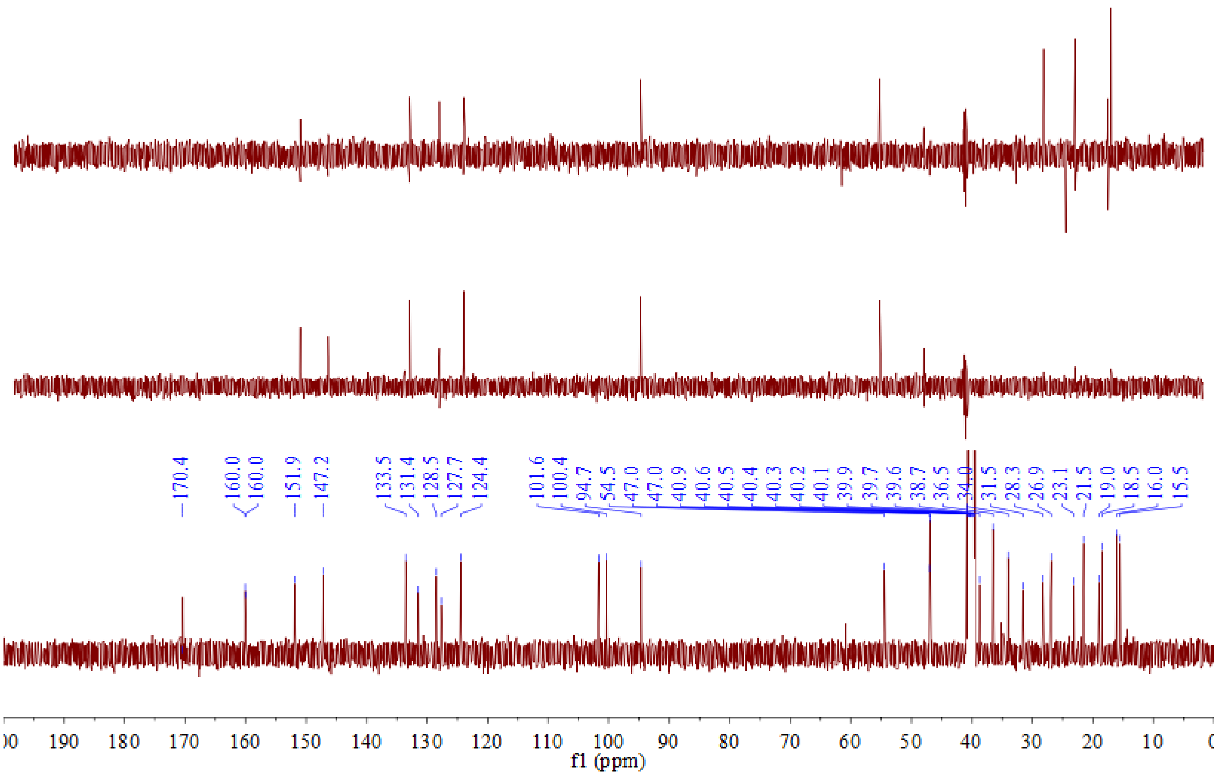

Figure S11. The ^{13}C NMR and DEPT spectrum (125 MHz) of **2** in DMSO-d_6

Elemental Composition Report **Page 1**

Single Mass Analysis
Tolerance = 1.0 mDa / DBE: min = -1.5, max = 50.0
Element prediction: Off
Number of isotope peaks used for i-FIT = 3

Monoisotopic Mass, Even Electron Ions
386 formula(e) evaluated with 1 results within limits (all results (up to 1000) for each mass)
Elements Used:
C: 0-100 H: 0-100 N: 0-5 O: 0-10
XUKUO
EN290-10 197 (3.302)

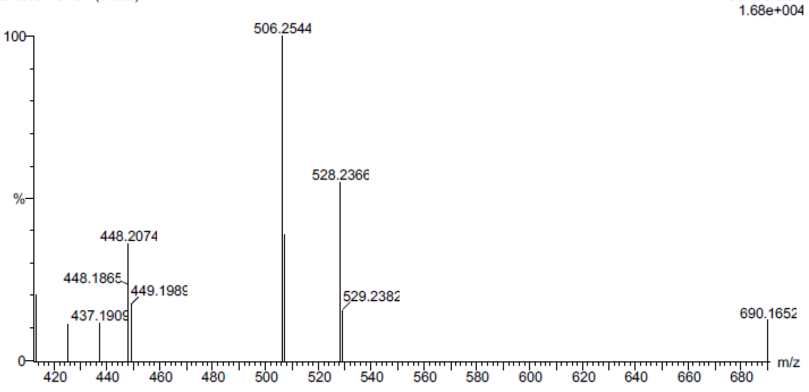

Minimum: -1.5
Maximum: 1.0 1.0 50.0

Mass	Calc. Mass	mDa	PPM	DBE	i-FIT	Norm	Conf(%)	Formula
506.2544	506.2543	0.1	0.2	13.5	28.8	n/a	n/a	C30 H36 N O6

Figure S12. The HR-ESI-MS data of **3**

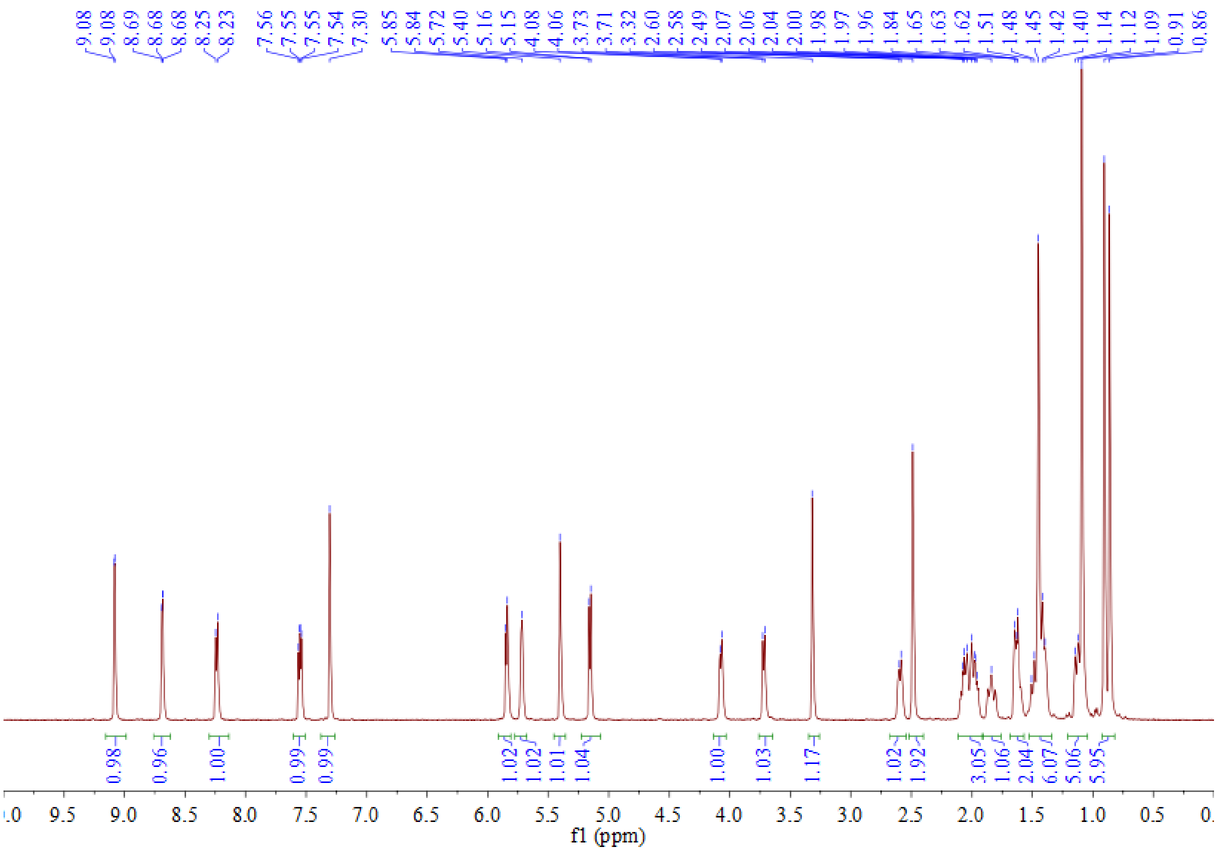

Figure S13. The ^{1}H NMR spectrum (500 MHz) of **3** in DMSO-d_6

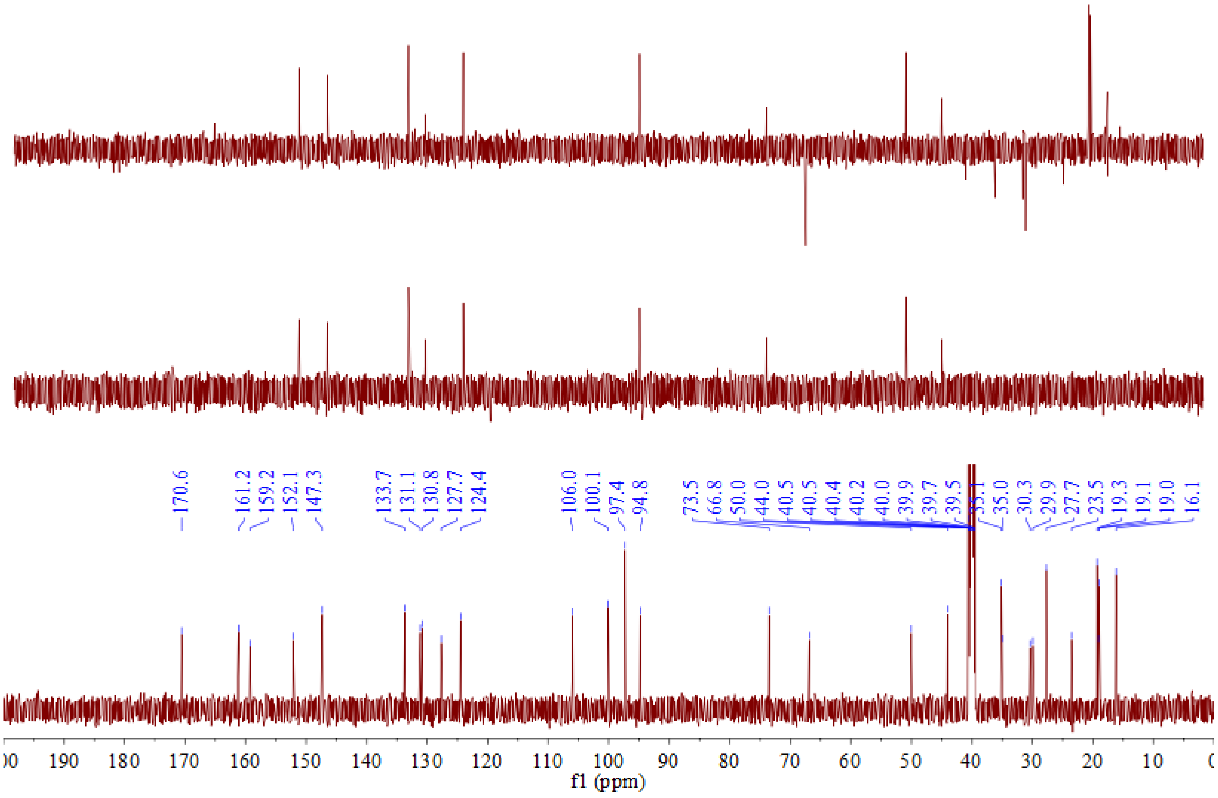

Figure S14. The ^{13}C NMR and DEPT spectrum (125 MHz) of **3** in DMSO-d_6

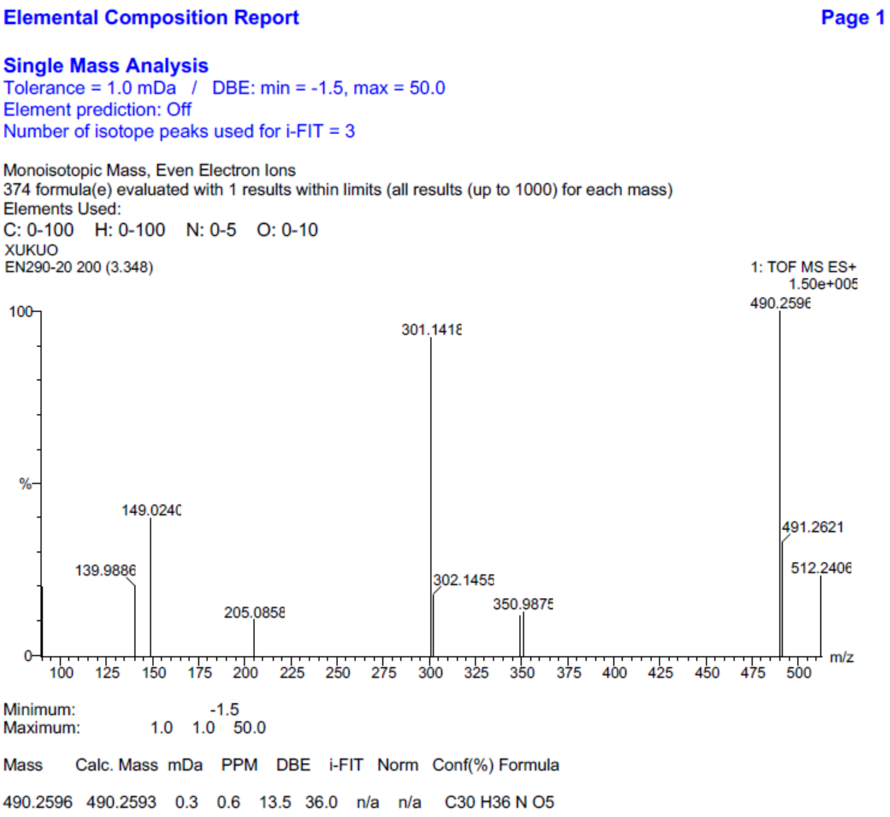

Figure S15. The HR-ESI-MS data of **4**

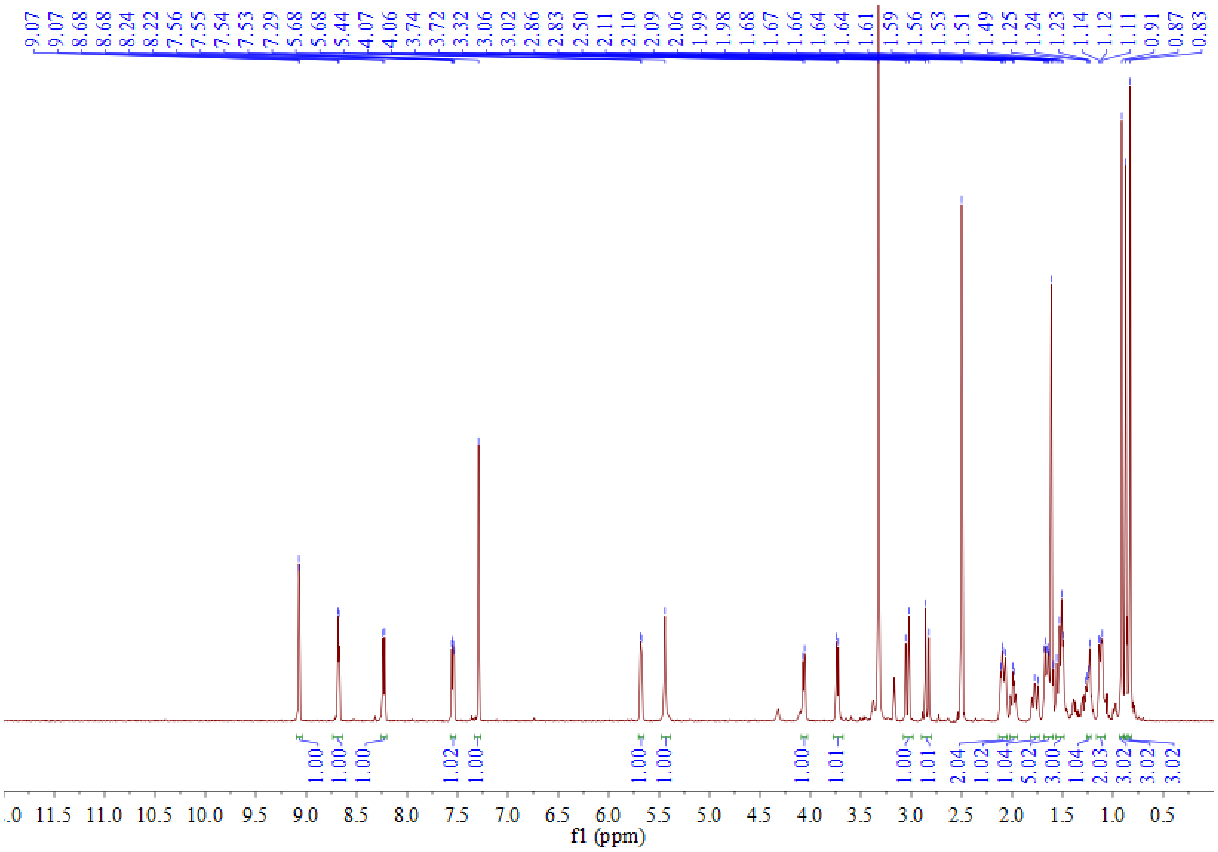

Figure S16. The ^{1}H NMR spectrum (500 MHz) of **4** in DMSO-d_6

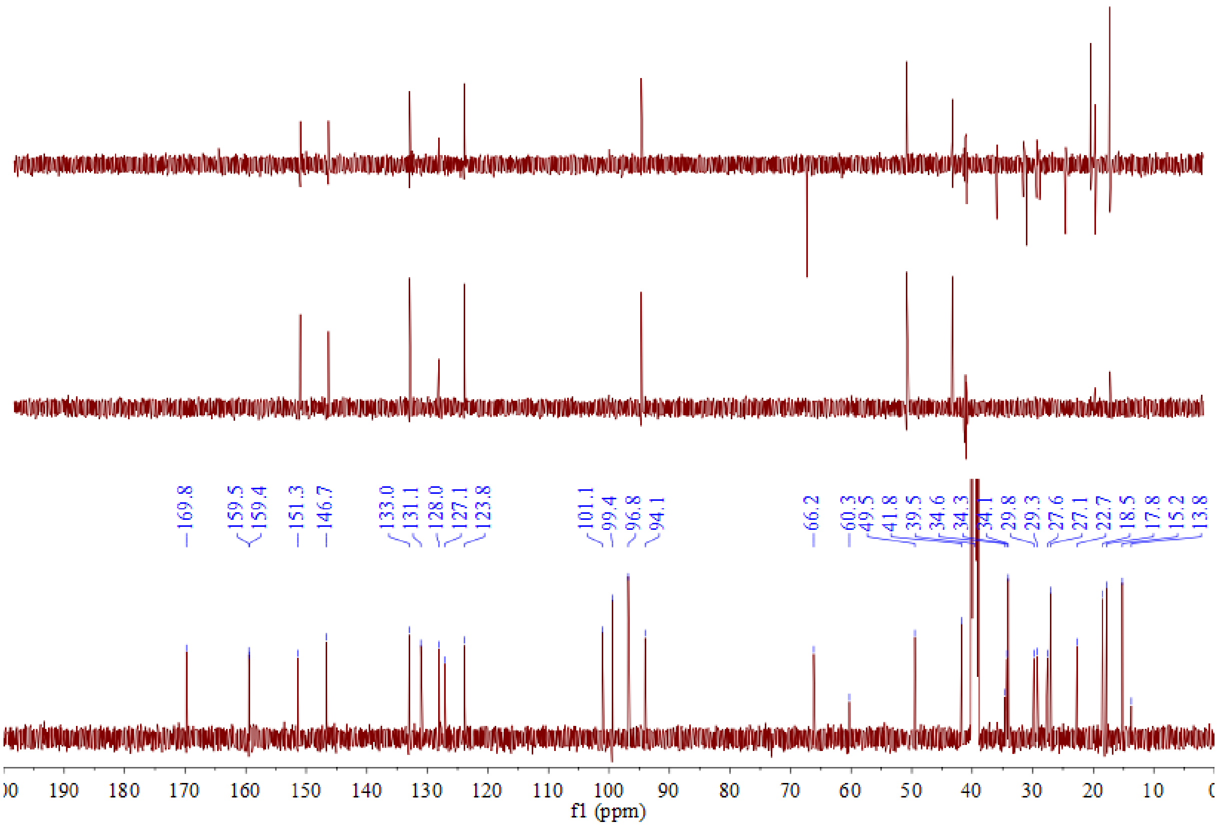

Figure S17. The ^{13}C NMR and DEPT spectrum (125 MHz) of **4** in DMSO-d_6

Elemental Composition Report **Page 1**

Single Mass Analysis
Tolerance = 1.0 mDa / DBE: min = -1.5, max = 50.0
Element prediction: Off
Number of isotope peaks used for i-FIT = 3

Monoisotopic Mass, Even Electron Ions
375 formula(e) evaluated with 1 results within limits (all results (up to 1000) for each mass)
Elements Used:
C: 0-100 H: 0-100 N: 0-5 O: 0-10
XUKUO
EN290-9-2 203 (3.399)

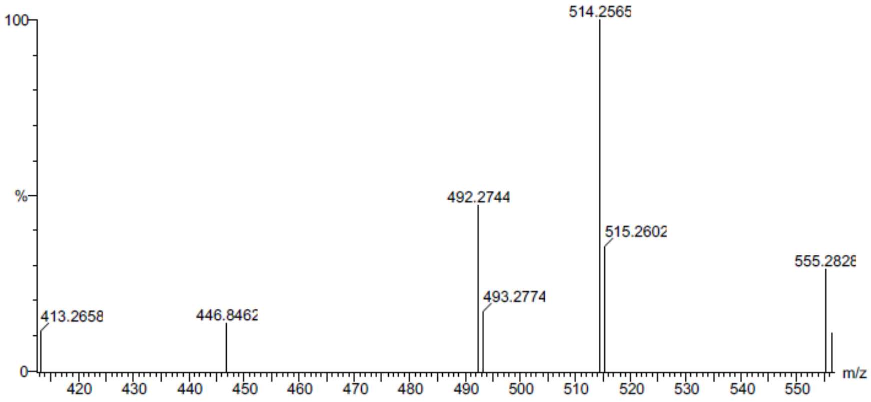

Minimum:			-1.5					
Maximum:		1.0	1.0	50.0				
Mass	Calc. Mass	mDa	PPM	DBE	i-FIT	Norm	Conf(%)	Formula
492.2744	492.2750	-0.6	-1.2	12.5	36.6	n/a	n/a	C30 H38 N O5

Figure S18. The HR-ESI-MS data of **5**

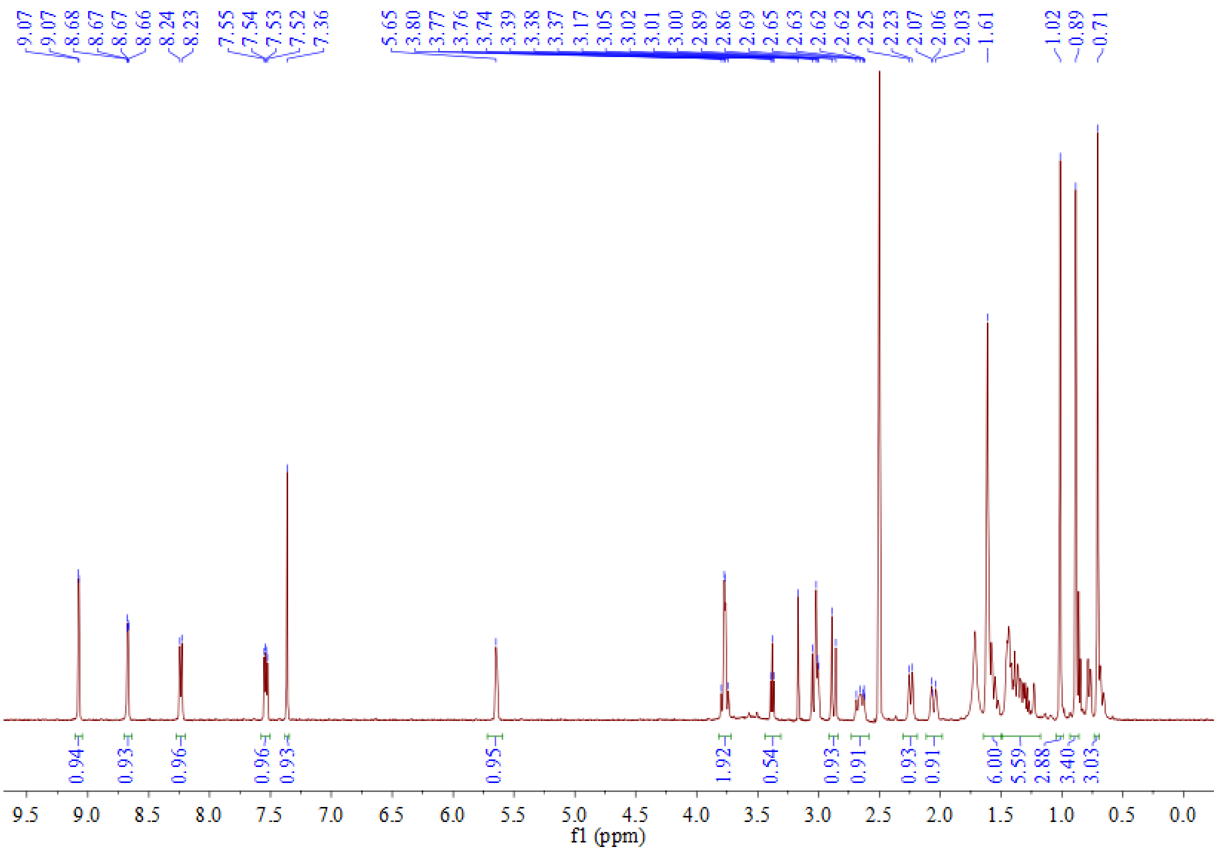

Figure S19. The ^{1}H NMR spectrum (500 MHz) of **5** in DMSO-d_6

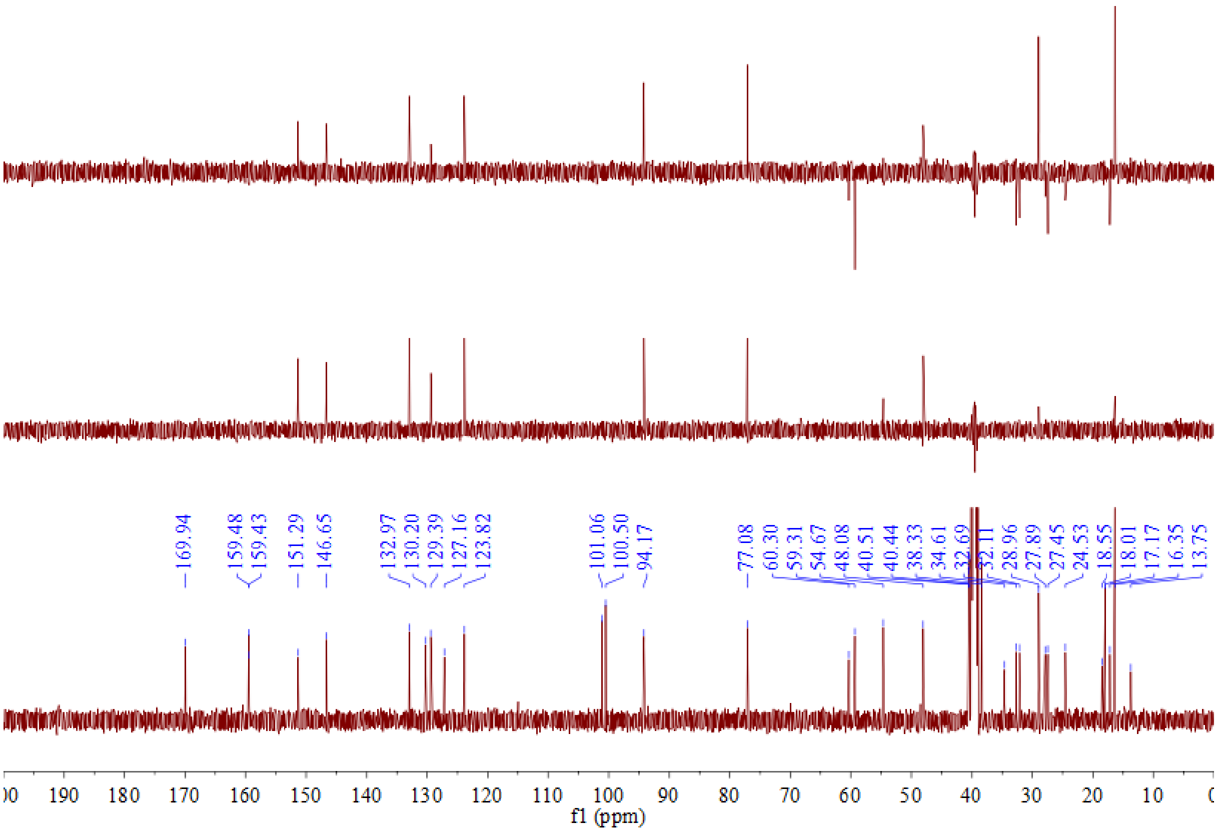

Figure S20. The ^{13}C NMR and DEPT spectrum (125 MHz) of **5** in DMSO-d_6

Elemental Composition Report **Page 1**

Single Mass Analysis
Tolerance = 1.0 mDa / DBE: min = -1.5, max = 50.0
Element prediction: Off
Number of isotope peaks used for i-FIT = 3

Monoisotopic Mass, Even Electron Ions
83 formula(e) evaluated with 1 results within limits (all results (up to 1000) for each mass)
Elements Used:
C: 0-50 H: 0-100 O: 0-10 Na: 0-1

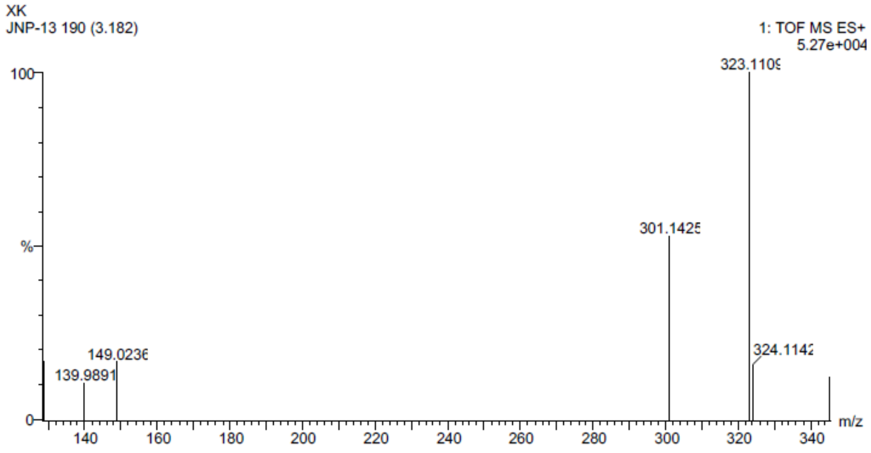

Minimum:				-1.5				
Maximum:		1.0	1.0	50.0				
Mass	Calc. Mass	mDa	PPM	DBE	i-FIT	Norm	Conf(%)	Formula
323.1109	323.1107	0.2	0.6	4.5	29.7	n/a	n/a	C14 H20 O7 Na

Figure S21. The HR-ESI-MS data of **6**

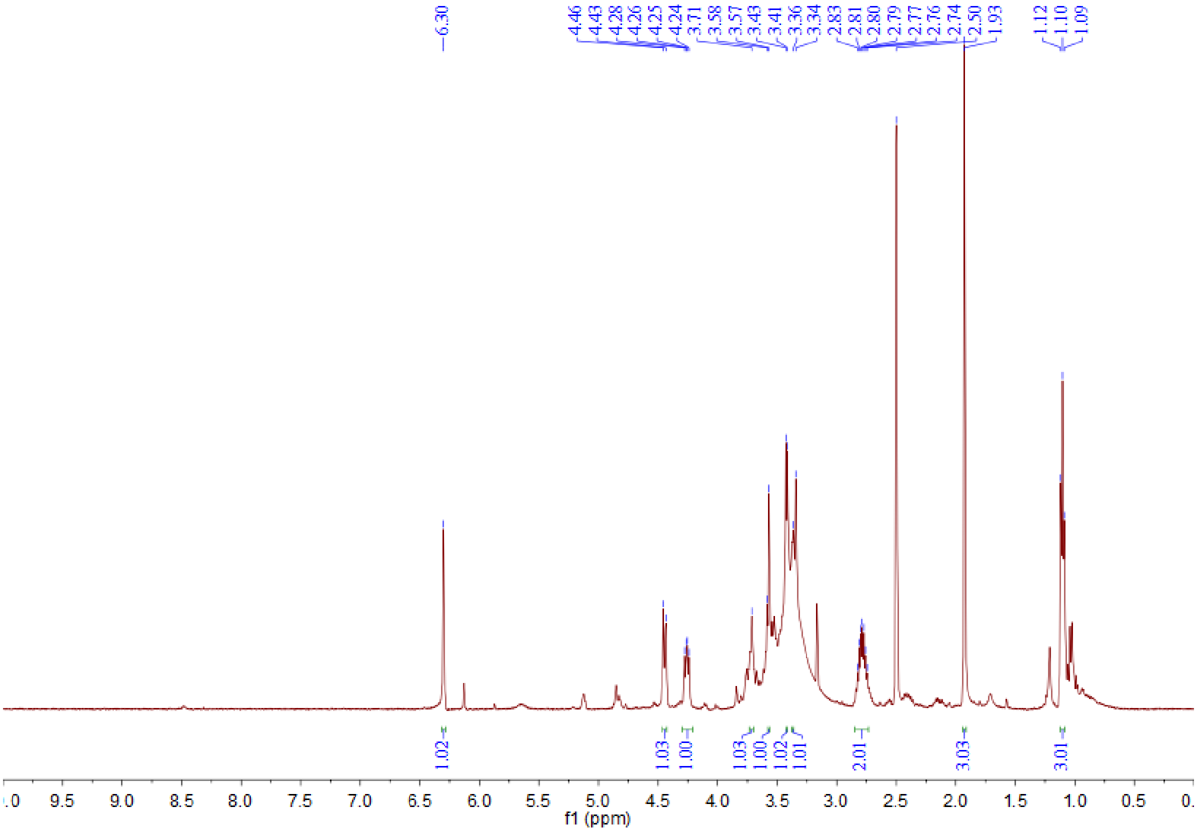

Figure S22. The ^{1}H NMR spectrum (500 MHz) of **6** in DMSO-d_6

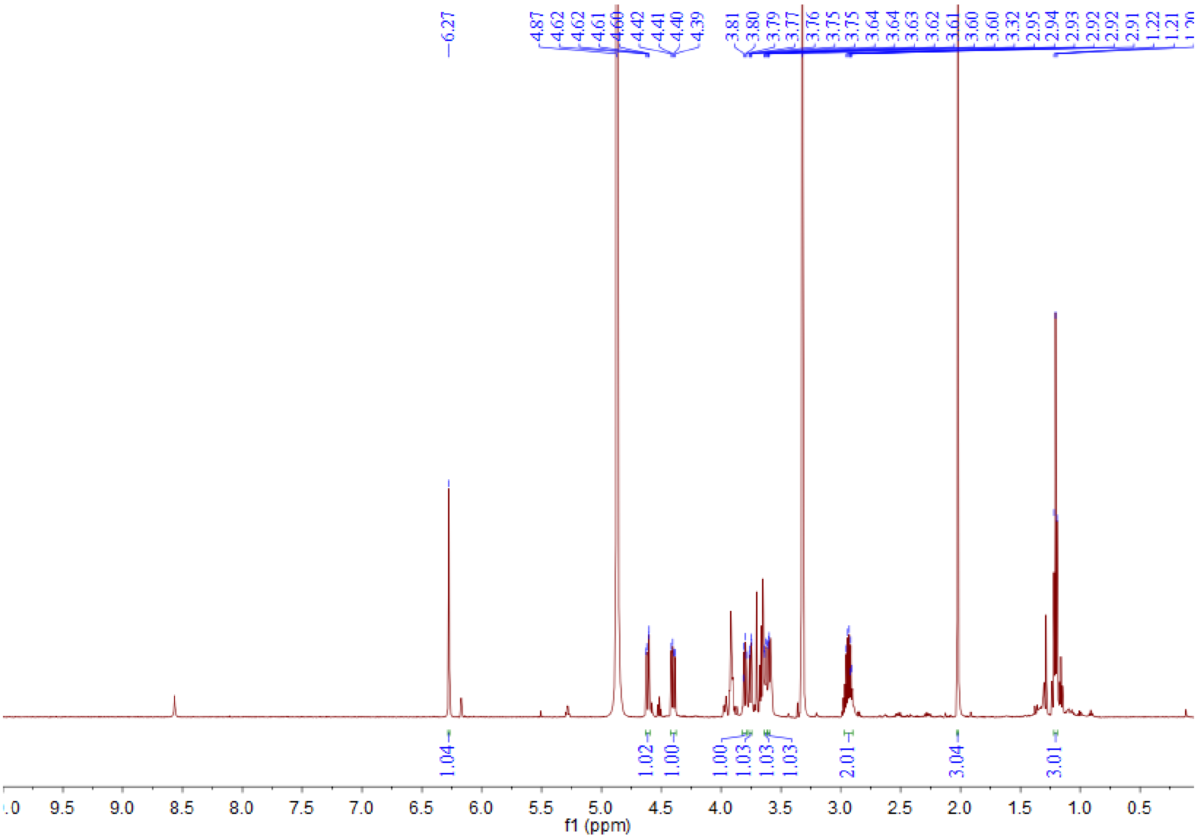

Figure S23. The ^{1}H NMR spectrum (500 MHz) of **6** in MeOH-d_4

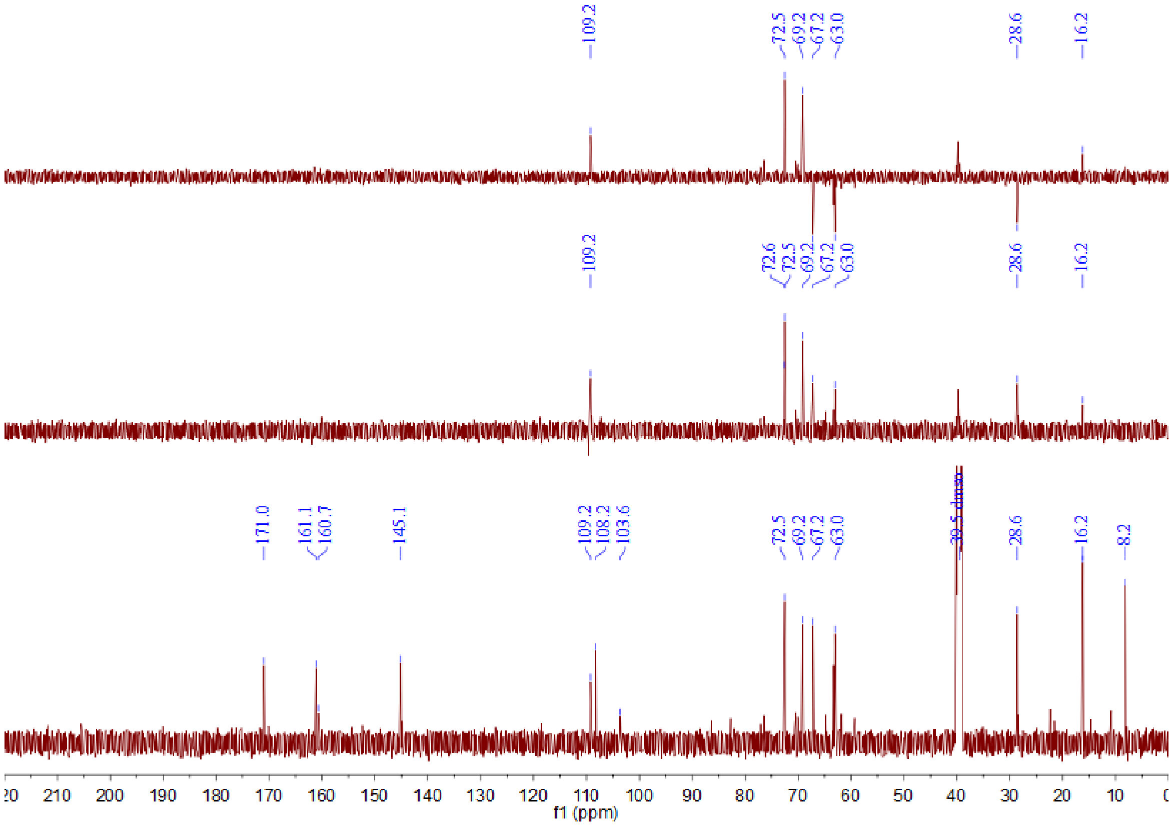

Figure S24. The ^{13}C NMR and DEPT spectrum (125 MHz) of **6** in DMSO-d_6

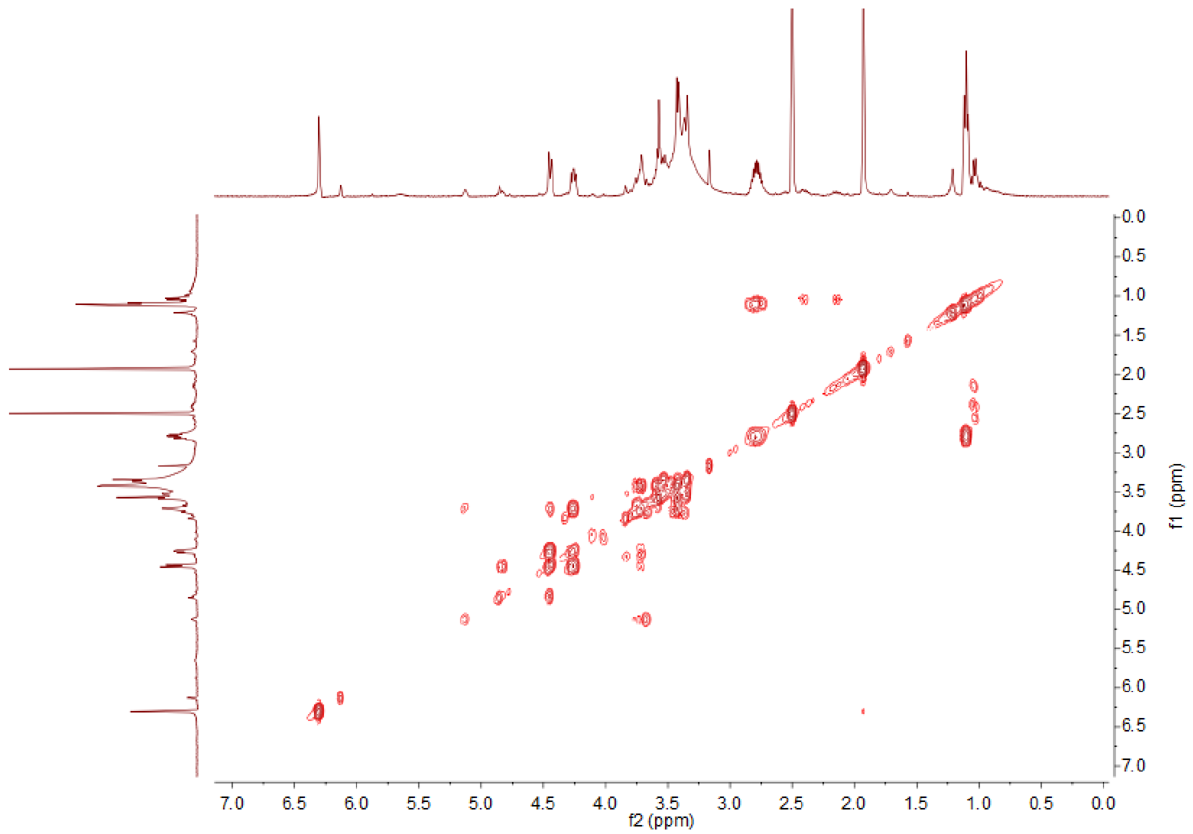

Figure S25. The ^{1}H-^{1}H COSY spectrum (500 MHz) of **6** in DMSO-d_6

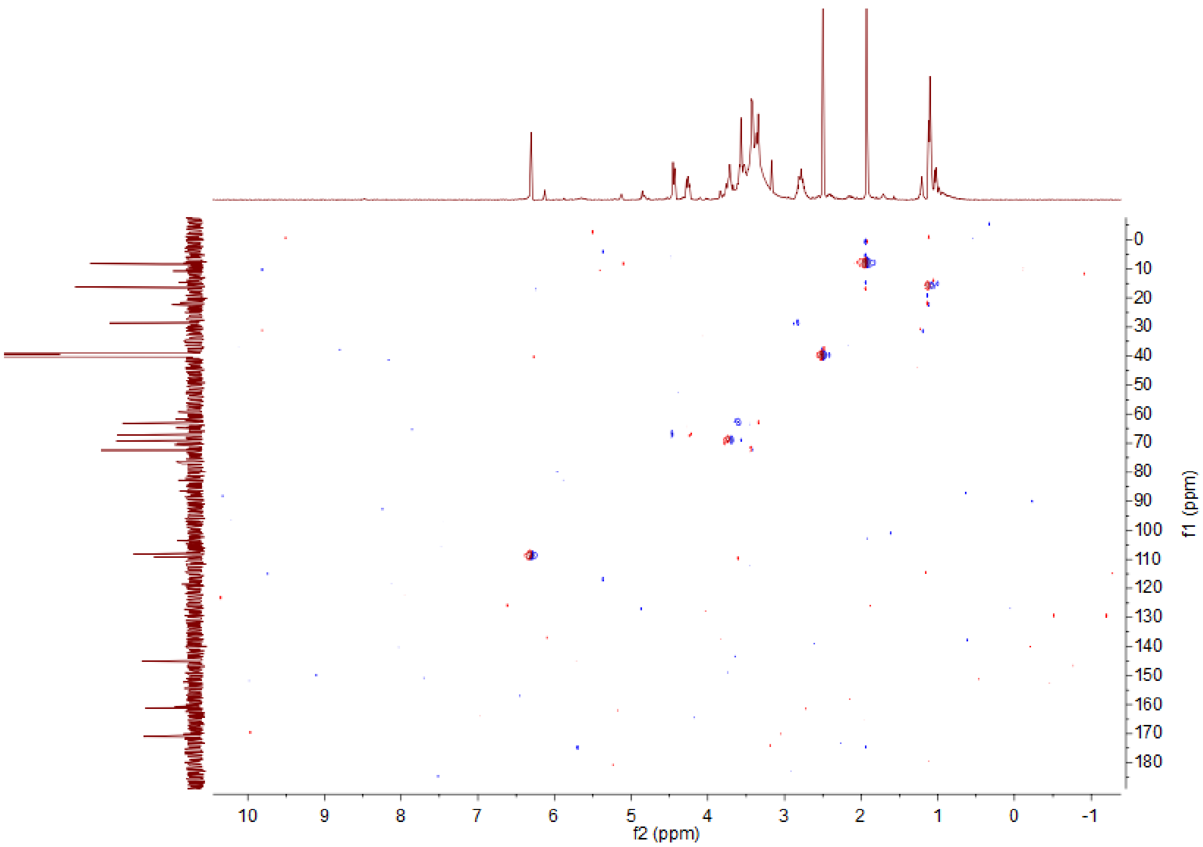

Figure S26. The HSQC spectrum (500 MHz) of **6** in DMSO-d_6

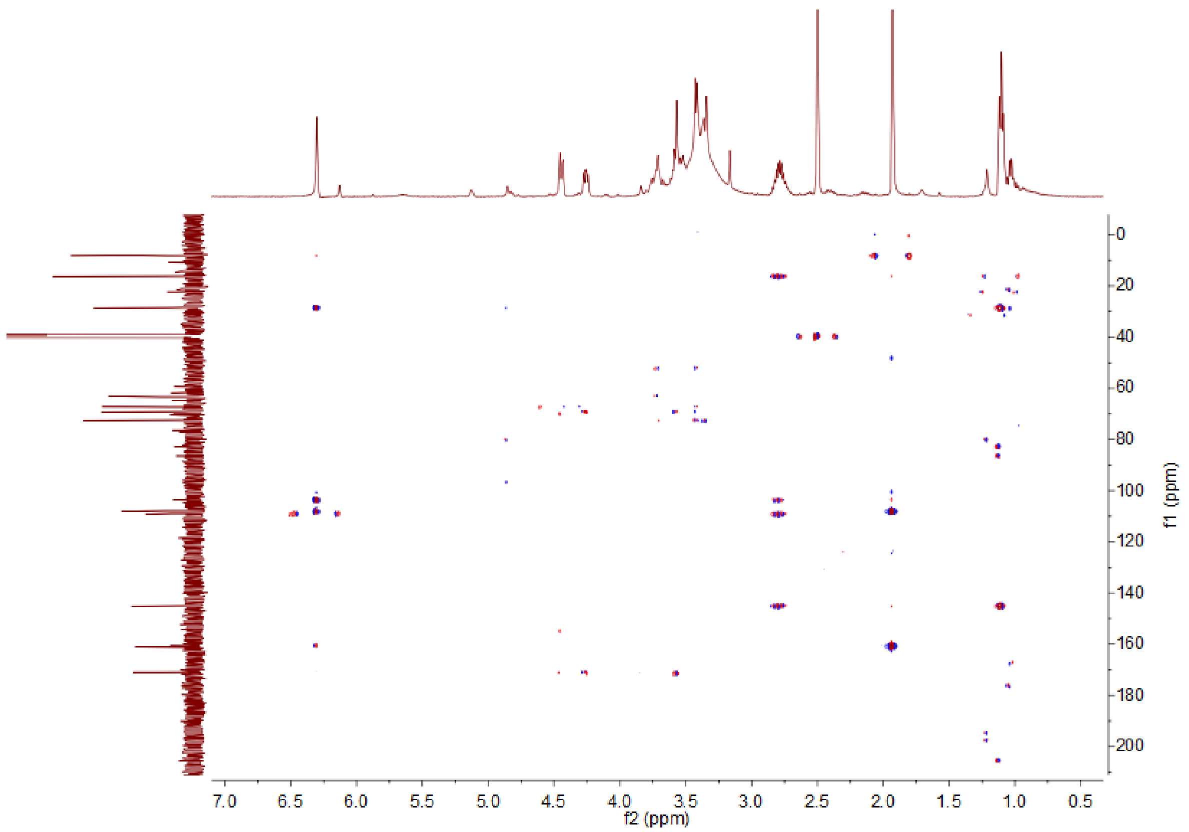

Figure S27. The HMBC spectrum (500 MHz) of **6** in DMSO-d_6

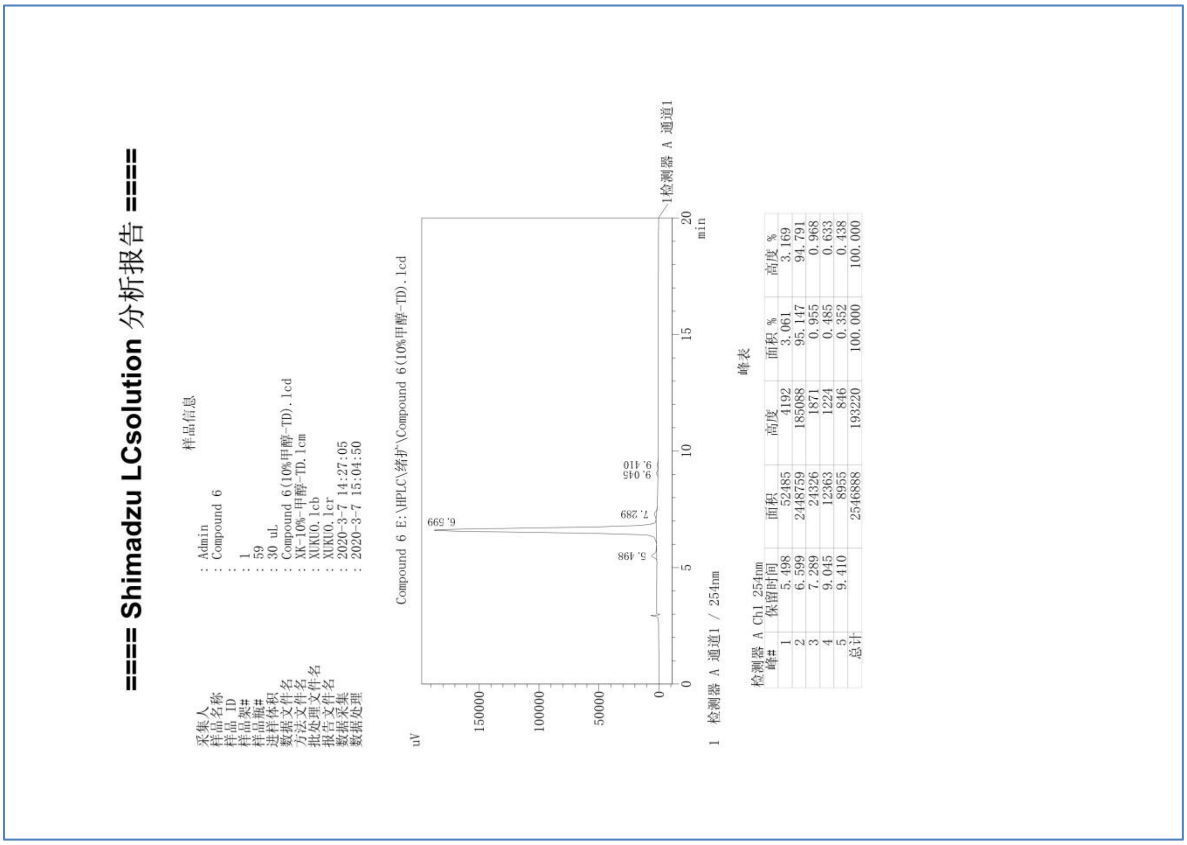

==== Shimadzu LCsolution 分析报告 ====

样品信息

采集人 : Admin
样品名称 : Compound 6
样品 ID :
样品架# : 1
样品瓶# : 59
进样体积 : 30 uL
数据文件名 : Compound 6(10%甲醇-TD).lcd
方法文件名 : XK-10%-甲醇-TD.lcm
批处理文件名 : XUKUO.lcb
报告文件名 : XUKUO.lcr
数据采集 : 2020-3-7 14:27:05
数据处理 : 2020-3-7 15:04:50

1 检测器 A 通道1 / 254nm

峰表

检测器 A Ch1 254nm

峰#	保留时间	面积	高度	面积 %	高度 %
1	5.498	52485	4192	3.061	3.169
2	6.599	2448759	185088	95.147	94.791
3	7.289	24326	1871	0.955	0.968
4	9.045	12363	1224	0.485	0.633
5	9.410	8955	846	0.352	0.438
总计		2546888	193220	100.000	100.000

Figure S28. The HPLC spectrum of **6** (254nm, 10-100%MeOH, 20 min)

Elemental Composition Report **Page 1**

Single Mass Analysis
Tolerance = 1.0 mDa / DBE: min = -1.5, max = 50.0
Element prediction: Off
Number of isotope peaks used for i-FIT = 3

Monoisotopic Mass, Even Electron Ions
72 formula(e) evaluated with 1 results within limits (all results (up to 1000) for each mass)
Elements Used:
C: 0-50 H: 0-100 O: 0-10 Na: 0-1
XK
JNP-14 170 (2.850) 1: TOF MS ES+ 1.18e+005

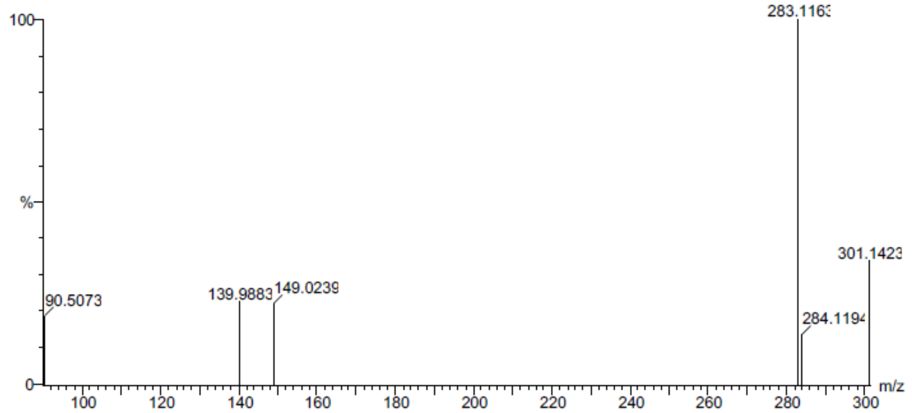

Minimum: -1.5
Maximum: 1.0 1.0 50.0

Mass	Calc. Mass	mDa	PPM	DBE	i-FIT	Norm	Conf(%)	Formula
283.1163	283.1158	0.5	1.8	2.5	32.3	n/a	n/a	C12 H20 O6 Na

Figure S29. The HR-ESI-MS data of **7**

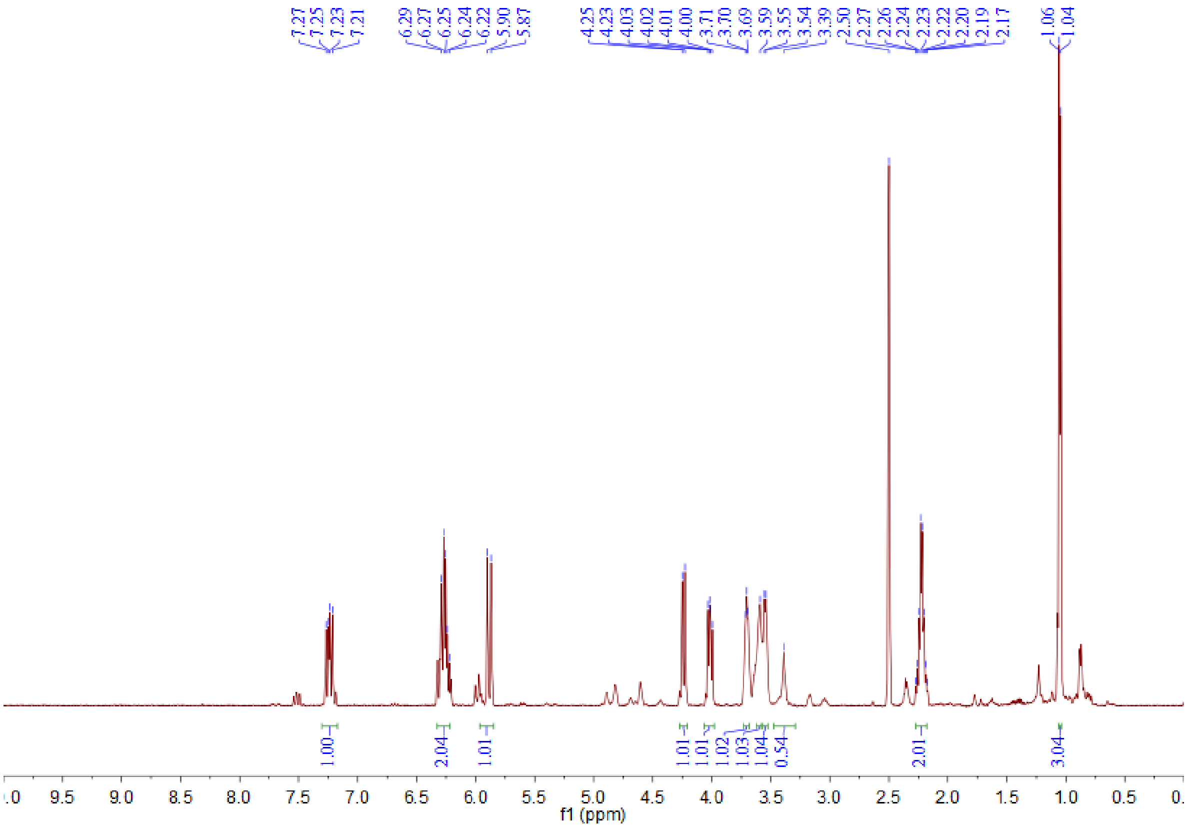

Figure S30. The ^{1}H NMR spectrum (500 MHz) of **7** in DMSO-d_6

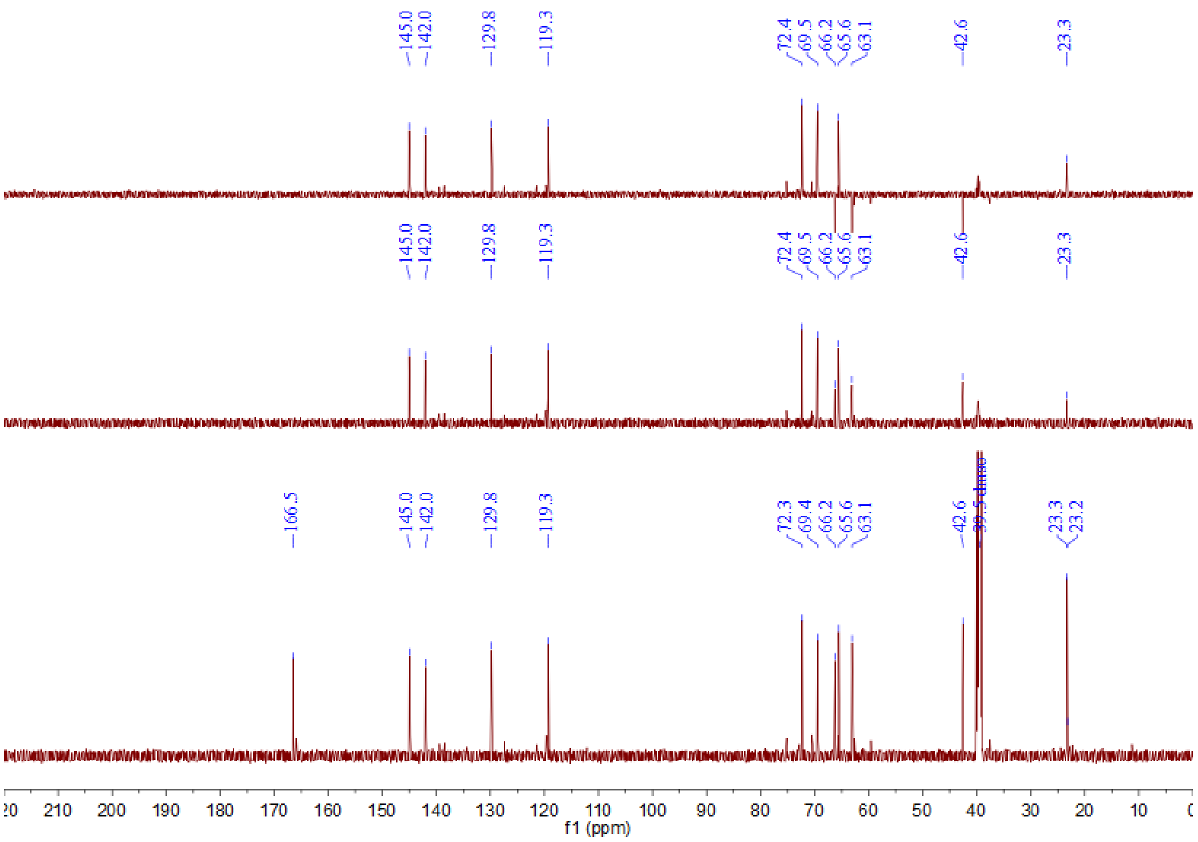

Figure S31. The ^{13}C NMR and DEPT spectrum (125 MHz) of **7** in DMSO-d_6

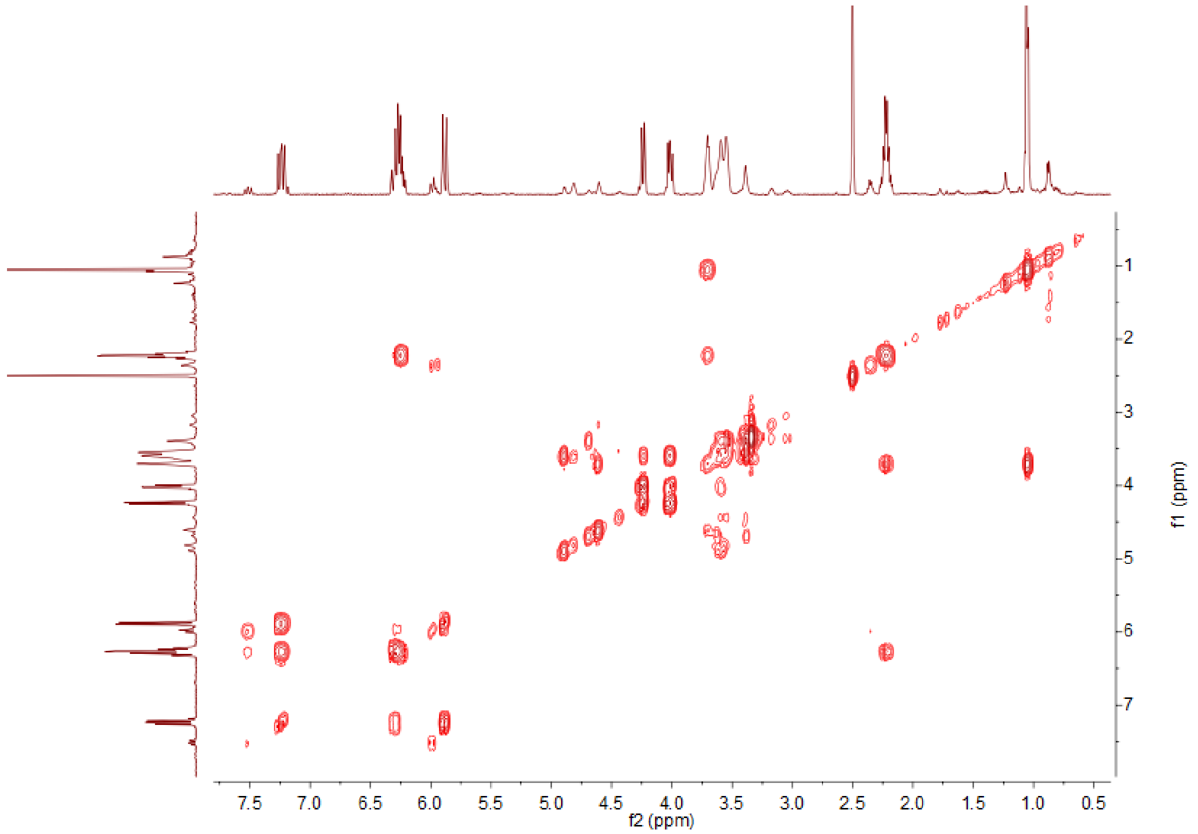

Figure S32. The ^{1}H-^{1}H COSY spectrum (500 MHz) of **7** in DMSO-d_6

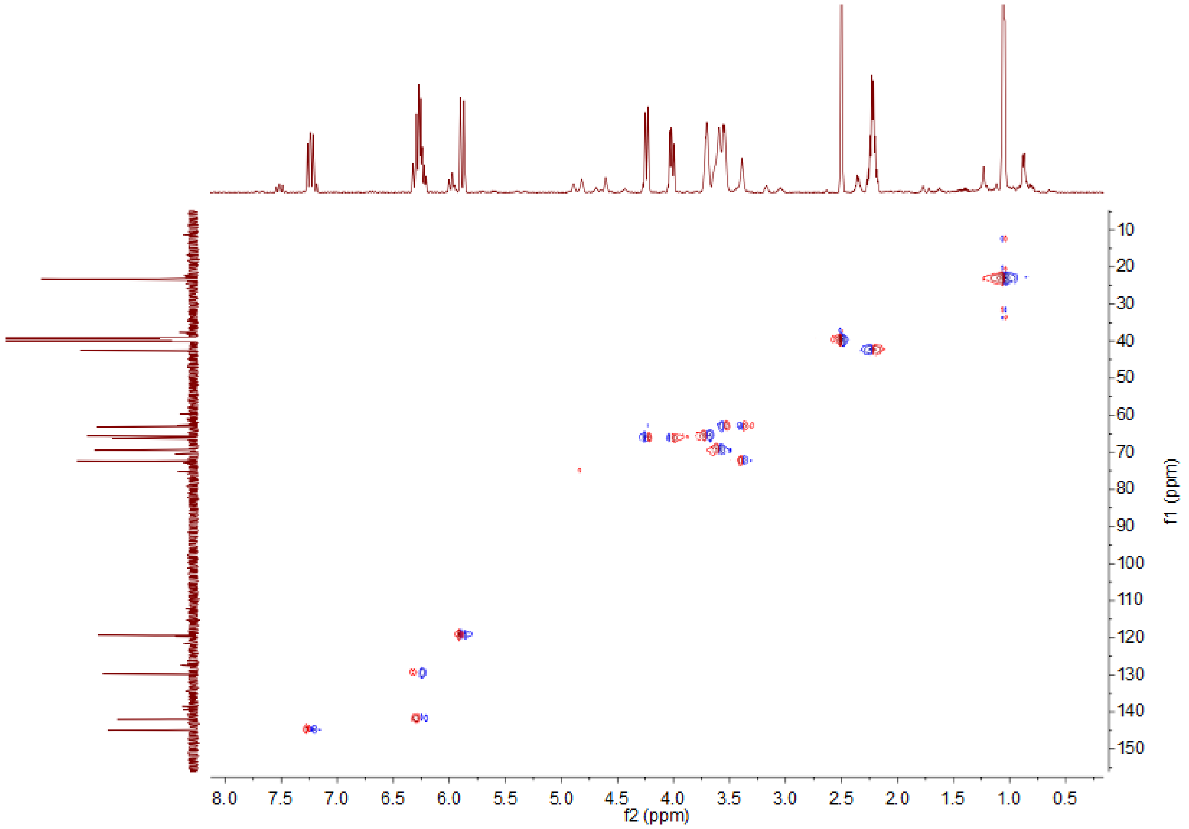

Figure S33. The HSQC spectrum (500 MHz) of **7** in DMSO-d_6

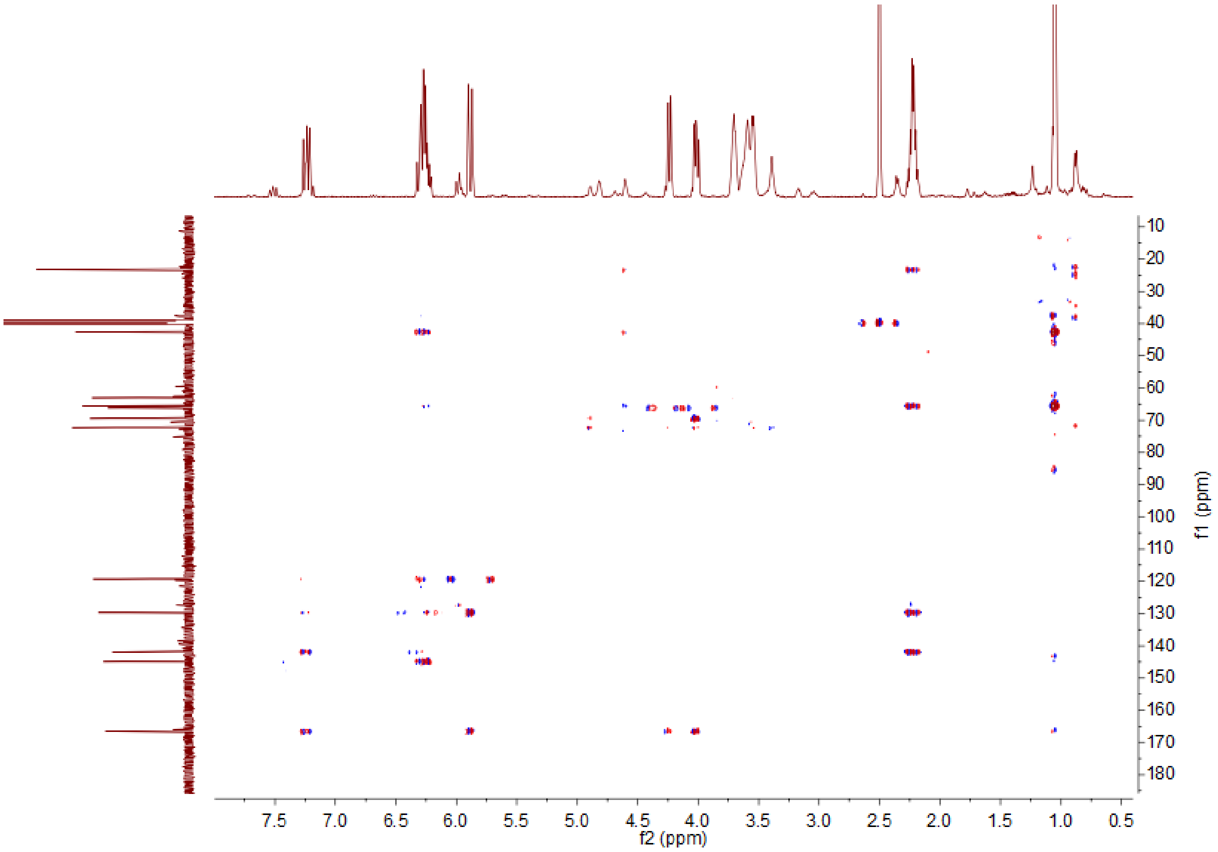

Figure S34. The HMBC spectrum (500 MHz) of **7** in DMSO-d_6

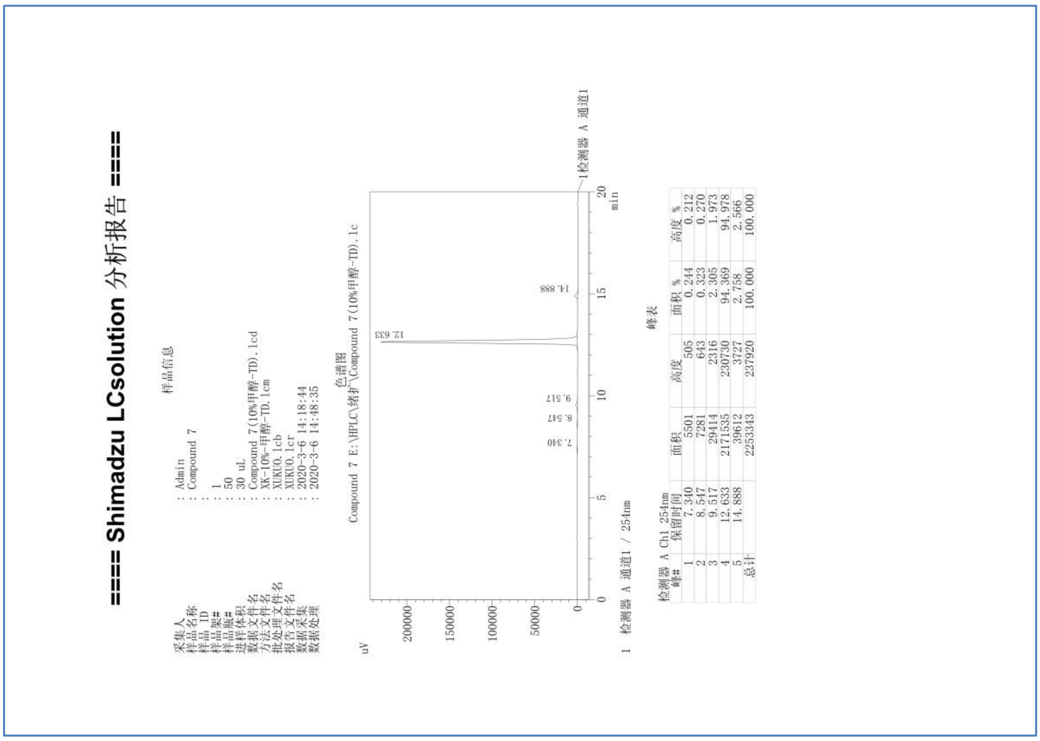

Figure S35. The HPLC spectrum of **7** (254nm, 10-100%MeOH, 20 min)

Theoretical calculations of compound 1

The theoretical calculations of ECD and NMR were carried on the Yinfo Cloud Platform (http://cloud.yinfotek.com). Conformational analysis of **1** was performed using Confab with systematic search algorithm at MMFF94 force field with RMSD threshold of 0.5 Å and energy window of 7 kcal/mol [1]. The energies of all dominative conformers were provided in Table S1. At first, conformers were optimized at PM6 and HF/6-31G(d) theory levels, consecutively. Room-temperature equilibrium populations were calculated according to Boltzmann distribution law, based on which dominative conformers were saved with the values over 1%. The chosen conformers were further optimized at B3LYP/6-31G(d) in gas phase. Vibrational frequency analysis confirmed the stable structures. Finally, the ECD calculations were executed in methanol with IEFPCM model using Time-dependent Density functional theory (TD-DFT) at the B3LYP/6-311G(d,p) level. Rotatory strengths for 50 excited states were calculated. The ECD spectrum of **1** was simulated by overlapping Gaussian functions (σ and UV-shift values were set 0.35 eV and –30 nm, respectively) [2]. As a result, the experimental ECD spectrum of **1** was matched well with the calculated spectrum of (7*E*, 12*S*, 16*S*, 17*R*, 20*R*, and 21*R*)-isomer.

The above optimized conformations were further conducted into ^{13}C-NMR calculations, which were carried out using the Gauge-Including Atomic Orbitals (GIAO) method at mPW1PW91/6-311+G(2d,p) level in DMSO simulated by the IEFPCM model [3]. Finally, the TMS-corrected NMR chemical shift values were averaged based on Boltzmann distribution and fitted to the experimental values by linear regression. As a result, the TMS-corrected computed ^{13}C-NMR chemical shift of compound **1** was fitted to the experimental values by Ordinary Least Squares (OLS) Linear Regression method in order to remove systematic error that results from the conformational search and random error from experimental conditions (Table S2). Relatively higher R^2 and lower CMAD and CLAD values were shown in both ^{13}C-NMR Linear Regression for (7*E*, 12*S*, 16*S*, 17*R*, 20*R*, and 21*R*)-isomer, which further supported the data of the ECD calculation.

Table S1. The selected conformers of **1** with Boltzmann distribution over 1%.

Conformers	Energy (kcal/mol)	Population (%)
1	108.64	29.29
2	108.69	26.92
3	108.91	18.57
4	108.92	18.26
5	110.23	2.00
6	110.31	1.75
7	110.44	1.40
8	110.47	1.33

Table S2. Experimental and calculated ^{13}C-NMR chemical shifts of **1**.

Position	Experimental	Calculated	Fitted	Residue
1	148.3	157.6497721	148.3272	-0.0272
2	128.5	139.5167951	130.8624	-2.36239
3	135	142.8913135	134.1126	0.887439
4	123.5	129.0017913	120.7348	2.765164
5	151.4	160.7459463	151.3093	0.090709
6	186.4	193.7836939	183.1297	3.270327
7	94	98.3128387	91.1767	2.823299
8	170.5	179.9479279	169.8037	0.696277
9	140.5	141.3593472	136.637	3.86296
10	164.3	172.2550605	162.3943	1.905679
11	148.4	169.691203	149.925	-1.5249
12	99	106.2241465	98.7965	0.203505
13	135.6	137.8683478	131.275	4.32532
14	127.8	141.0855288	132.373	-4.5733
15	23.6	31.06219489	26.40409	-2.80409
16	41.5	44.55883742	39.40341	2.096587
17	40.4	49.48447924	44.14756	-3.74756
18	25.3	29.42959099	24.83164	0.468355
19	27.6	30.48711538	25.8502	1.749798
20	75.1	88.64159996	79.86183	-4.76183
21	44	53.25734315	47.7814	-3.7814
22	25.8	30.98632834	26.33102	-0.53102
23	29.7	34.43590228	29.65348	0.046515
24	173.6	183.4828274	173.2084	0.39163
25	67	74.17570582	67.92897	-0.92897
26	19.5	21.48233474	17.17723	2.322773
27	18.1	21.29209459	16.994	1.106004
28	150.5	159.124968	149.748	0.751958

29	114.3	124.6640714	116.557	-2.25695
30	21.7	22.50753193	18.16465	3.535352

References:

1. Noel M OBoyle, Tim V, ermeersch, Christopher J Flynn, Anita R Maguire Maguire, and Geoffey R Hutchison. Confab-systematic generation of diverse low-energy conformers. *Journal of Cheminformatics*, 2011, 3, 3–8.
2. Stephens, P. J.; Harada, N. ECD cotton effect approximated by the Gaussian curve and other methods. *Chirality* 2010, 22, 229–233.
3. Michael W. Lodewyk, Matthew R. Siebert, and Dean J. Tantillo. Computational Prediction of 1H and 13C Chemical Shifts: A Useful Tool for Natural Product, Mechanistic, and Synthetic Organic Chemistry. *Chem. Rev.*, 2012, 112, 1839–1862.

Antimicrobial assay

Antifungal bioassay: The isolated compounds were tested in vitro for the antifungal activity against five pathogenic fungi: *Alternaria alternata* (*A. alternata*), *Botrytis cinerea* (*B. cinerea*), *Fusarium oxysporum* (*F. oxysporum*), *Penicillium digitatum* (*P. digitatum*), and *Valsa mali* (*V. mali*). All of these phytopathogenic fungi tested were purchased from Qingdao Agricultural University (Qingdao, People's Republic of China). Antifungal activity was assessed by the microbroth dilution method in 96-well flat-microtiter plates using potato dextrose (PD) medium. The test compounds were made up to 2 mg/mL in DMSO. The commercial fungicide prochloraz was used as a positive control. The solution of equal concentration of DMSO was used as a negative control. The fungi were incubated in the PD medium for 18−36 h at 28 ± 0.5 ℃ at 150 rpm, and spore concentrations of different microorganism were diluted to approximately 1×10^6 colony-forming units/mL (CFU/mL) with PD medium. Test compounds (10 μL) were added to 96-well microplates, and 90 μL of PD medium was added. Serial dilutions were made in the 96-well round-bottom sterile plates in triplicate in 50 μL of PD medium, and then 50 μL of the fungal suspension was added. In flat-microtiter plates, tested compounds, fungal suspension, and sterile water were added to make up final concentrations of the compounds in the range of 1−64 μg/mL. Each measurement consisted of three replicates. Cultures then grew in the dark at 28 ± 0.5 ℃ for 48 h. Minimum inhibitory concentrations (MICs) were inspected as the lowest concentrations in which no fungal growth could be observed.

Antibacterial bioassay: The isolated compounds were tested in vitro for the antibacterial activity against four pathogenetic bacteria: *Escherichia coli* (*E. coli*), *Micrococcus luteus* (*M. luteus*), *Pseudomonas aeruginosa* (*P. aeruginosa*), and *Ralstonia solanacearum* (*R. solanacearum*). Antibacterial activity was assessed by the microbroth dilution method in 96-well flat-microtiter plates using a beef protein liquid (BP) medium. Antibacterial activity was assessed according to the same procedure as antifungal bioassay. Chloromycetin was used as a positive control, and the solution of equal concentration of DMSO was used as a negative control. Cultures were grown for 24 h at 37 ± 0.5 ℃ in the dark without shaking, in a moist chamber.

Table S3. ^{1}H, ^{13}C NMR COSY and HMBC data of compound **1** in DMSO-d_6.

Position	δ_H (mult, *J* in Hz)	δ_C, type	COSY	HMBC
2	8.89 (s)	148.3, CH		4, 7,
3		128.5, C		
4	8.14 (d, 8.0)	135.0, CH	5	2, 6, 7
5	7.50 (m)	123.5, CH	4, 6	3
6	8.66 (d, 4.5)	151.4, CH	5	4
7		186.4, C		
9		164.3, C		
10		140.5, C		
11		170.5, C		
12	7.09 (s)	94.0, CH		3, 10
14		99.0, C		
15	7.32 (s)	148.4, CH		9, 11, 16, 20
16		135.6, C		
17	5.75 (s)	127.8, CH	18	14, 19, 30
18a	2.29 (overlap)	23.6, CH_2	17, 19	16, 20, 24
18b	2.06 (d, 18.0)			
19	2.57 (dd, 5.0, 12.0)	41.5, CH	18	14, 17, 21, 25, 29
20		40.4, C		
21a	1.89 (t, 12.5)	25.3, CH_2	22	14, 19, 23, 31
21b	0.77 (d, 12.5)			
22a	2.18 (t, 14.0)	27.6, CH_2	21	20, 24, 32
22b	1.31 (d, 14.0)			
23		75.1, C		
24		44.0, C		
25a	2.27 (overlap)	25.8, CH_2	26	19, 23, 27, 29
25b	1.45 (d, 15.5)			
26a	2.42 (dt, 14.0, 5.0)	29.7, CH_2	25	24
26b	2.20 (overlap)			
27		173.6, C		
29	4.43 (s)	67.0, CH_2		19, 23, 25
30	1.36 (s)	19.5, CH_3		14, 17
31	0.94 (s)	18.1, CH_3		14, 19, 20
32		150.5, C		
33a	5.08 (s)	114.3, CH_2		23, 34
33b	4.84 (s)			
34	1.75 (s)	21.7, CH_3		23, 33

Table S4. ^{1}H, ^{13}C NMR COSY and HMBC data of compound **6** in DMSO-d_6.

**Position**	**δ_H (mult, *J* in Hz)**	**δ_C, type**	**COSY**	**HMBC**
1a	4.44 (d, 11.0)	67.2, CH_2	2	3, 7′
1b	4.26 (dd, 11.0, 7.0)			
2	3.71 (m)	69.2, CH	1, 3	4
3	3.42 (overlap)	72.5, CH	2, 4	1
4a	3.58 (m)	63.0, CH_2	3	2
4b	3.36 (overlap)			
1′		103.6, C		
2′		161.1, C		
3′		108.2, C		
4′		160.7, C		
5′	6.30 (s)	109.2, CH		1′, 3′, 9′
6′		145.1, C		
7′		171.0, C		
8′	1.93 (s)	8.2, CH_3		2′, 4′
9′	2.78 (m)	28.6, CH_2	10′	1′, 5′
10′	1.10 (t, 7.5)	16.2, CH_3	9′	6′

Table S5. ^{1}H, ^{13}C NMR COSY and HMBC data of compound **7** in DMSO-d_6.

**Position**	**δ_H (mult, *J* in Hz)**	**δ_C, type**	**COSY**	**HMBC**
1a	4.24 (d, 11.0)	66.2, CH_2	2	3, 1′
1b	4.01 (dd, 11.0, 7.5)			
2	3.59 (m)	69.4, CH	1, 3	4
3	3.38 (overlap)	72.3, CH	2, 4	1
4a	3.55 (m)	63.1, CH_2	3	2
4b	3.39 (overlap)			
1′		166.5, C		
2′	5.88 (d, 15.5)	119.3, CH	3′	4′
3′	7.24 (dd, 15.5, 10.0)	145.0, CH	2′, 4′	1′, 5′
4′	6.27 (overlap)	129.8, CH	3′, 5′	2′, 6′
5′	6.25 (overlap)	142.0, CH	4′, 6′	3′, 7′
6′	2.22 (m)	42.6, CH_2	5′, 7′	4′, 8′
7′	3.70 (m)	65.6, CH	6′, 8′	5′
8′	1.05 (d, 6.0)	23.3, CH_3	7′	6′

Table S6. The experimental and reported specific rotations of compounds **2**−**5**.

**Compounds**	**Experimental specific rotations/**$[\alpha]_D^{25}$	**Reported specific rotations/**$[\alpha]_D^{25}$	**Ref.**
**2**	+71 °(*c* 0.1, CH_2Cl_2)	+58 °(*c* 0.1, CH_2Cl_2)	[1]
**3**	+149 °(*c* 0.1, CH_2Cl_2)	+124 °(*c* 0.2, CH_2Cl_2)	[2]
**4**	+137 °(*c* 0.1, CH_2Cl_2)	+167 °(*c* 0.2, CH_2Cl_2)	[1]
**5**	+85 °(*c* 0.2, CH_2Cl_2)	+104 °(*c* 0.1, CH_2Cl_2)	[3]

References

[1] Li, C.; Gloer, J.B.; Wicklow, D.T.; Dowd, P.F. Antiinsectan decaturin and oxalicine analogues from *Penicillium thiersii*. *J. Nat. Prod.* **2005**, *68*, 319–322.

[2] Zhang, Y.; Li, C.; Swenson, D.C.; Gloer, J.B.; Wicklow, D.T.; Dowd, P.F. Novel antiinsectan oxalicine alkaloids from two undescribed fungicolous *Penicillium* spp. *Org. Lett.* **2003**, *5*, 773–776.

[3] Wang, P.L.; Li, D.Y.; Xie, L.R.; Wu, X.; Hua, H.M.; Li, Z.L. Novel decaturin alkaloids from the marine-derived fungus *Penicillium oxalicum*. *Nat. Prod. Commun.* **2013**, *8*, 1397–1398.
